# Biomass-burning organic aerosols as a pool of atmospheric reactive triplets to drive multiphase sulfate formation

**DOI:** 10.1073/pnas.2416803121

**Published:** 2024-12-13

**Authors:** Zhancong Liang, Liyuan Zhou, Yuqing Chang, Yiming Qin, Chak K. Chan

**Affiliations:** ^a^Division of Physical Science and Engineering, King Abdullah University of Science and Technology, Thuwal 23955-6900, Saudi Arabia; ^b^School of Energy and Environment, City University of Hong Kong, Hong Kong, China

**Keywords:** air pollution, wildfire, multiphase chemistry, photosensitization, brown carbon

## Abstract

Biomass-burning organic aerosol(s) (BBOA) are rich in brown carbon, which significantly absorbs solar irradiation and potentially accelerates global warming. Despite its importance, the multiphase photochemistry of BBOA after light absorption remains poorly understood due to challenges in determining the oxidant concentrations and the reaction kinetics within aerosol particles. In this study, we explored the photochemical reactivity of BBOA particles in multiphase S(IV) oxidation to sulfate. We found that sulfate formation in BBOA particles under light is predominantly driven by photosensitization involving the triplet excited states (^3^BBOA^*^) instead of iron, nitrate, and S(IV) photochemistry. Rates in BBOA particles are three orders of magnitude higher than those observed in the bulk solution, primarily due to the fast interfacial reactions. Our results highlight that the chemistry of ^3^BBOA^*^ in particles can greatly contribute to the formation of sulfate, as an example of the secondary pollutants. Photosensitization of BBOA will likely become increasingly crucial due to the intensified global wildfires.

## Significance

Biomass-burning organic aerosol(s) (BBOA) not only significantly impact Earth’s climate by absorbing solar radiation but also generate excited triplet states (^3^BBOA^*^) that react with other atmospheric molecules, known as photosensitization. Understanding the photosensitization processes is increasingly important due to the intensifying wildfires worldwide. In this study, we demonstrate the high reactivity of ^3^BBOA^*^ in aerosol particles, indicated by the rapid sulfate formation via SO_2_ oxidation. Notably, the photosensitized sulfate formation in BBOA particles occurs much more rapidly than in diluted solutions, which is attributable to prominent interfacial reactions. These findings suggest that multiphase photosensitization in BBOA can drive the rapid formation of secondary pollutants and may exacerbate air quality degradation, particularly for regions prone to wildfires.

Organic aerosols resulting from wildfires and open biomass burning, collectively termed biomass-burning organic aerosol(s) (BBOA), represent the predominant source of global primary organic aerosols (POA). Annually, BBOA contributes about 60 to 85% of the total POA, with emissions estimated at approximately 31 to 36 Tg (1). These aerosols are rich in brown carbon (BrC) that can significantly absorb solar radiation, accelerating the warming of the Earth–atmosphere system (2).

**Fig. 1.**
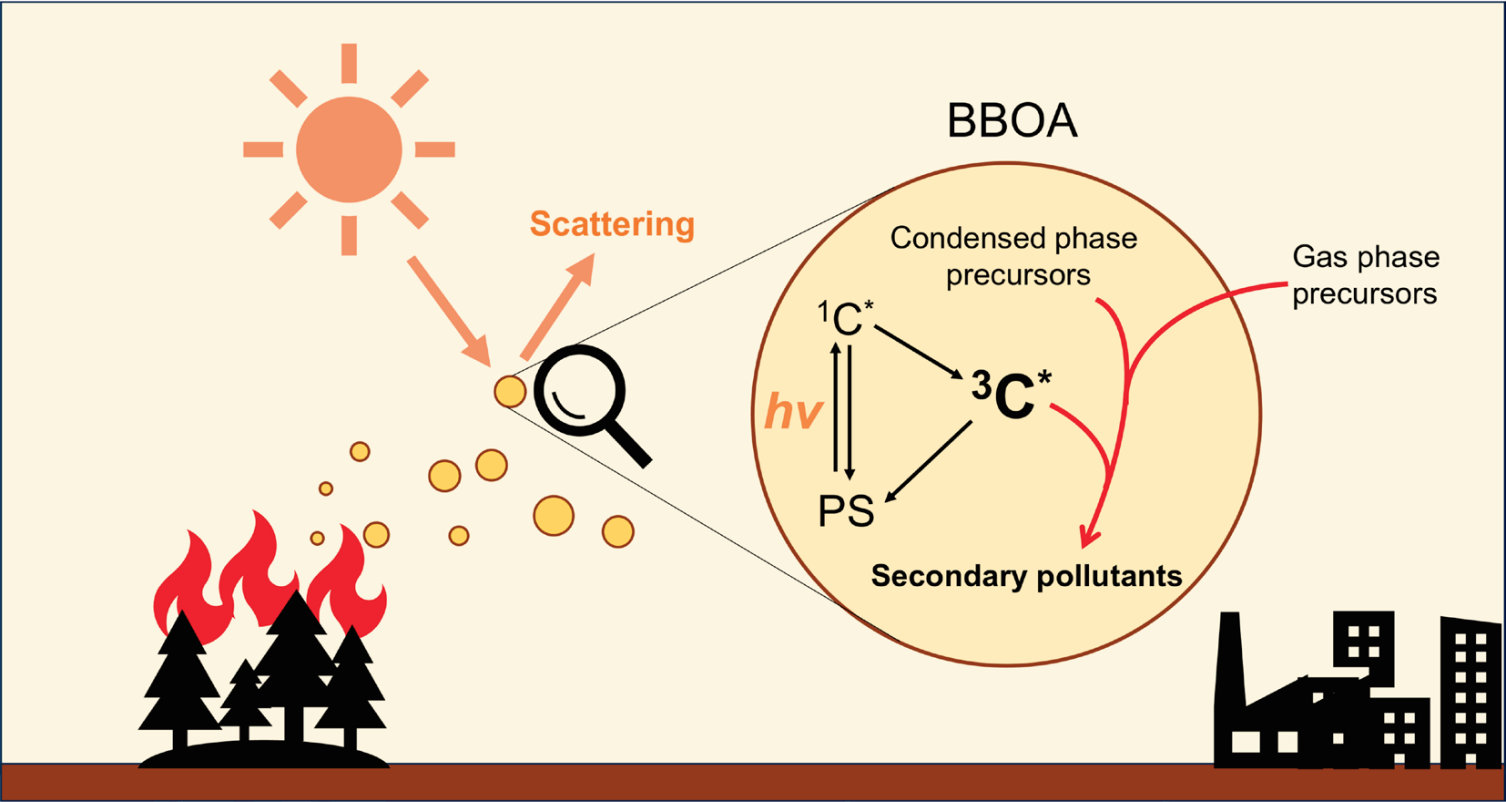
The photosensitization processes in BBOA. This diagram illustrates the key steps in the photosensitization process within BBOA. The black arrows indicate the self-cycling of photosensitizers (PS). The arrow from the photosensitizer to ^1^C^∗^ depicts the excitation of molecules from ground states to singlet excited states upon photon absorption. Subsequent arrows from ^1^C^*^ to PS represent the relaxation of singlet states through both reactive (e.g., photolysis) and nonreactive (e.g., fluorescence and internal conversion) pathways. The arrow from ^1^C^∗^ to ^3^C^∗^ denotes intersystem crossing to triplet states, while the final arrow from ^3^C^∗^ to PS shows the relaxation pathways for triplet states, including phosphorescence and triplet–triplet annihilation. The red arrow linking condensed and gas phase precursors to ^3^C* illustrates the multiphase reactions driven by ^3^C^*^ to form secondary particulate pollutants (i.e., photosensitization).

The light-absorption properties and atmospheric persistence of BrC have garnered extensive research interest (3–7). It is critical to recognize that the fundamental process underlying light absorption involves the transition of electrons between energy states. Excited states with high energy will be generated upon the photon absorption by a BrC molecule. Recent studies indicate that BrC, upon excitation to singlet states (i.e., ^1^C^*^), can transition to longer-lived triplet states (i.e., ^3^C^*^) via intersystem crossing (ISC), besides undergoing photolysis and nonreactive relaxation processes such as fluorescence (Fig. 1). Triplets have significantly longer lifetimes than their singlet counterparts (8, 9), which enables reactions with various molecules (referred to as substrates) at appreciable rates to form secondary pollutants, known as photosensitization (10–15).

Sulfate aerosols, an important secondary air pollutant, are predominantly produced by multiphase SO_2_ oxidation during haze episodes (16). However, field measurements of sulfate formation rates often greatly exceed predictions from models based on conventional gas-phase and in-cloud reaction schemes. While multiphase SO_2_ oxidation has been proposed as a potential explanation for this discrepancy, the detailed mechanisms and kinetics remain less understood (17, 18). Previous studies have noted the presence of sulfate in BB particles, especially those transported through urban and industrial areas with elevated SO_2_ levels (19–21). Liu et al. (22) observed an excess of sulfate beyond that expected from chloride depletion during the aging of BB plumes, suggesting additional sulfate formation pathways. Notably, recent studies have reported enhanced sulfate production in both incense-burning particles and model biomass-burning photosensitizer (PS) particles under light illuminations, compared to those in the dark (23–25). The sulfate formation in illuminated photosensitizer particles prevails over that in photolyzing nitrate particles (25), a potentially important pathway for sulfate formation (26, 27). This highlights the role of photochemical processes initiated by the triplets of BBOA (i.e., ^3^BBOA^*^) beyond the direct climate impacts of BBOA, particularly as the frequency and intensity of wildfires are expected to rise globally due to climate change (28–30).

Despite these findings, the aerosol-phase reaction kinetics involving ^3^C^*^ and atmospheric substrates, such as SO_2_, remain poorly understood. One of the main challenges is the transient nature of ^3^C^*^ which complicates direct measurements (31). The decay of a chemical probe with known reaction rate constants with ^3^C^*^ was the only existing approach to determine the steady-state [^3^C^*^] in diluted solutions. However, the probe decay is subjected to back electron donation and light screening in concentrated aerosols, known as inhibition, which causes great underestimation (32, 33). The kinetic data, largely derived from dilute solutions using single-model photosensitizers, may not accurately reflect the complex interplays within BBOA, which contains a diverse mix of photosensitizers and substrates (34, 35). Moreover, increasing evidence has shown that the kinetics for multiphase reactions in concentrated aerosols with high solute strength can differ greatly from those in the bulk solutions (16, 36–39). Excess quenchers are typically used to ascertain the potential importance of ^3^C^*^, through observation of suppressed reactant decay or product formation. However, the quencher can also react with other oxidants and generate by-products, introducing uncertainties (23, 40, 41). Overall, the lack of adequate kinetic evaluations has significantly limited our understanding of the capability of ^3^BBOA^*^ to drive multiphase reactions and exacerbate air pollution.

Herein, we reported a kinetic study on the multiphase reactivity involving ^3^BBOA^*^ in submicron aerosol particles, using an aerosol flow reactor (AFT) coupled with multiple offline analytical tools. In this work, we referred to the water extract of the BB particles as BBOA. We focused on the water extract due to its better compatibility with analytical tools for deeper insights into our study. The potential effects of water-insoluble components were also discussed. We focused on sulfate formation from SO_2_ oxidation as an indicator of the multiphase reaction kinetics. This choice was driven by the atmospheric significance of sulfate and the relatively straightforward mechanism compared to that of organic oxidations. We used chemical probes and transient absorption spectroscopy (TA) to determine the oxidant concentrations and reaction rate constants in diluted solutions and then extrapolate to aerosol conditions, enabling modeling of the sulfate formation rate and comparisons with the measurement results. Mass spectrometry provides identification of the potential photosensitizers in BBOA. In addition, quantum chemical calculation and electron paramagnetic resonance spectroscopy (EPR) connect the reactivities of ^3^BBOA^*^ to photochemical parameters such as redox potentials, broadening our mechanistic understanding of BBOA-driven reactions. The overall goal of this work is to provide quantitative insights into the photosensitizing capabilities of BBOA particles and facilitate future parameterization.

## Results

### Sulfate Formation Kinetic in Illuminated BBOA Particles.

**Fig. 2.**
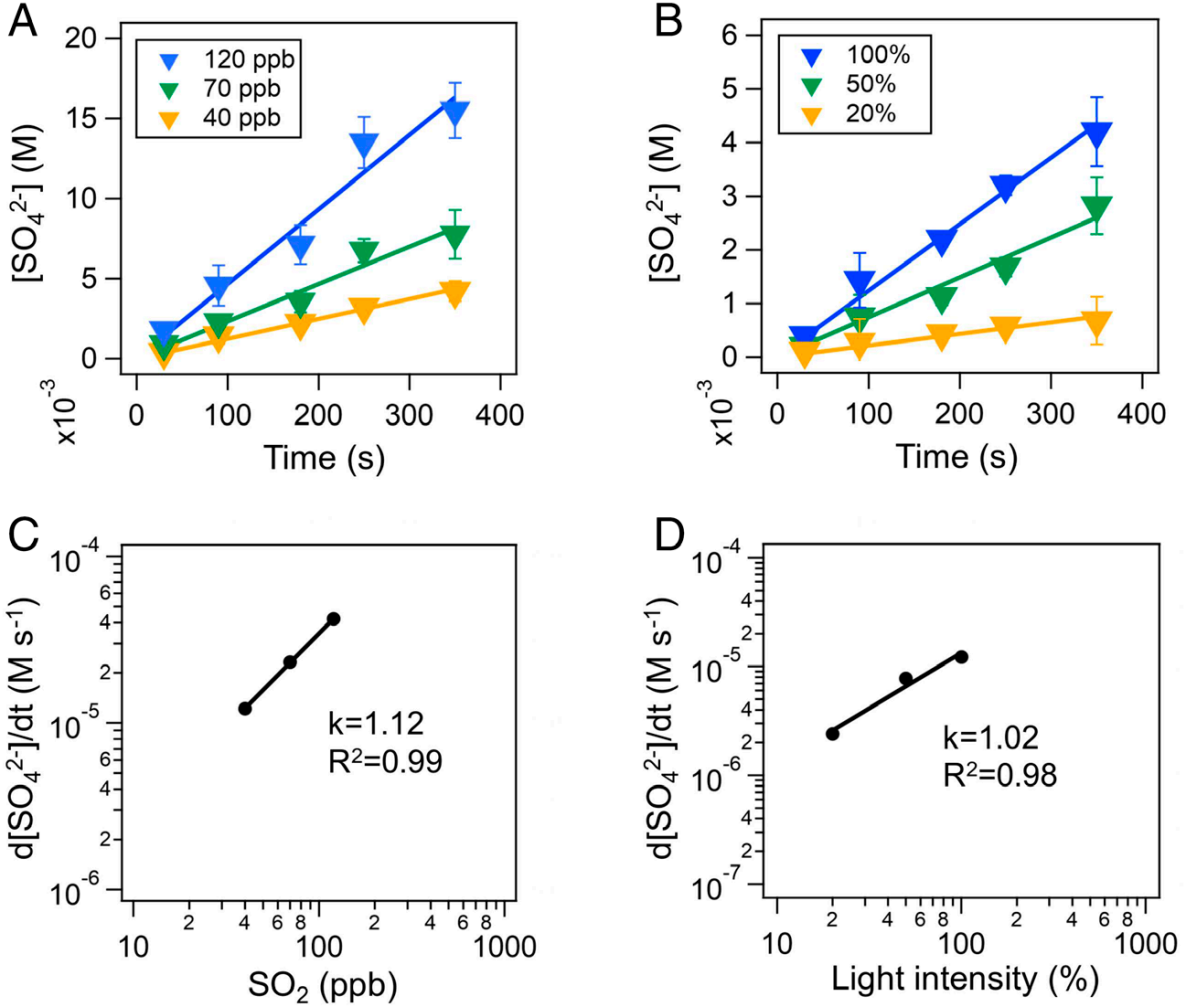
(*A*) Sulfate concentrations in BBOA as a function of illumination time at different SO_2_ concentrations. The light intensity of 100% indicates the full flux from light tubes; (*B*) Sulfate concentrations in BBOA as a function of illumination time at different light intensities. The light intensities of 50% and 20% were achieved by using neutral density filters for light attenuation. The SO_2_ concentration is 40 ppb; (*C*) The dependence of sulfate formation rate on SO_2_ concentrations, the light intensity is 100%; (*D*) The dependence of sulfate formation rate on the light intensity, the SO_2_ concentration is 40 ppb. All experiments were under N_2_ conditions and at 80% RH. The initial sizes of BBOA particles are all 100 nm.

Fig. 2 shows the kinetics of sulfate formation in the BBOA particles (atomized from the water extract of BB particles) under different SO_2_ concentrations and light intensities. The sulfate concentration in BBOA linearly increases with reaction time under all conditions, with faster rises observed at higher SO_2_ concentrations and light intensities, suggesting photochemical oxidation of SO_2_ (Fig. 2 *A* and *B*).

In contrast, experiments conducted in the dark with SO_2_ exposure showed negligible sulfate formation, indicating minimal contribution from nonphotochemical pathways (*SI Appendix*, Fig. S1). SO_2_ at the air–water interface could undergo forbidden excitation to its triplet state (i.e., ^3^SO_2_^*^), followed by hydrolysis to hydroxyl radical (i.e., OH•) capable of oxidizing SO_2_ (42, 43). To determine whether ^3^SO_2_^*^ photochemistry contributes to the observed sulfate formation in BBOA under light, we quantified the sulfate formation in nonphotoactive organic buffer droplets (Sodium malonate/Sodium bimalonate = 1:1) at the same [SO_2_], light intensity, initial particle size, and comparable pH (around 4, will be discussed later). The liquid water content of the buffer droplet is higher than that of BBOA, which allows more S(IV) dissolution. Malonate is less reactive than phenolic compounds toward OH•, thus it is not expected to suppress the [OH•] in buffer droplets compared with BBOA. However, negligible sulfate formation in organic buffer droplets under illumination was found, suggesting that ^3^SO_2_^*^ chemistry plays a minor role (*SI Appendix*, Fig. S1). The production of oxidants via photolysis of iron salts, iron organic complexes, and nitrate is possible, but their roles in the sulfate formation were also deemed minor, as doubling the iron and nitrate concentrations in BBOA only resulted in less than 5% increases in sulfate formation (*SI Appendix*, Fig. S2). Consequently, the dominant pathway for sulfate formation in illuminated BBOA is likely through the oxidation of S(IV) by ^3^BBOA^*^. In addition to SO_2_, effective photosensitized conversions of organic sulfur compounds to sulfate have been reported in the aquatic environments and likely sea-spray aerosols (44–46). A recent study by Rao et al. (47) suggested that such conversions can take place at the interface of microdroplets even in the absence of light. Nevertheless, no sulfate formation was observed in illuminated BBOA particles in the absence of SO_2_, indicating organic sulfur compounds, if present, were not a significant source of sulfate in BBOA particles (*SI Appendix*, Fig. S1).

Using TA, the quenching rate constant of ^3^BBOA^*^ by S(IV) [k_^3^BBOA^∗^, S(IV)_] was determined to be 6.0 × 10^8^, 5.1 × 10^8^, and 3.2 × 10^8^ M^–1^ s^–1^ at pH of 1, 4, and 7, respectively (*SI Appendix*, Fig. S3). Thermodynamic models suggested that the pH of BBOA particles ranges from 3 to 5 (48). Using a combined method of pH indicator paper and RGB analysis (*SI Appendix*, Fig. S4 and Text S1), we estimated the pH of BBOA particles to be around 4, which is consistent with the estimate based on the charge-conservation rule (49). At this pH, HSO_3_^−^ dominates the dissolved S(IV) species (*SI Appendix*, Fig. S3). ^3^BBOA^*^ may react with HSO_3_^−^ via energy transfer, electron transfer, and hydrogen transfer. While the energy transfer from ^3^BBOA^*^ to S(IV) can also lead to ^3^S(IV)^*^ and ultimately OH•, probe experiments indicate that the OH• concentration would be too low (e.g., 10^−18^ M) to compete with ^3^BBOA^*^ for oxidizing S(IV) (*SI Appendix*, Text S2), partially due to the low redox potential of ^3^BBOA^*^ to abstract electrons from water to yield OH• (will be discussed later).

We propose the reactions between ^3^BBOA^*^ and S(IV) via electron transfer and hydrogen transfer, illustrated as follows using HSO_3_^−^ as an example:

[R1] HSO3−+B3BOA∗→HSO3•+BBOA•−,

[R2] HSO3−+B3BOA∗→SO3•−+HBBOA•.

Subsequent self-reactions of the sulfite radicals and reactions with HSO_3_^−^ will lead to sulfate formation (50). The ^3^BBOA^*^ concentration increases with light intensity. The observed slopes for the sulfate formation rate vs. SO_2_ concentration and light intensity are 1.12 and 1.02, respectively, on the log–log plots (Fig. 2 *C* and *D*), suggesting that the reaction kinetics between ^3^BBOA^*^ and S(IV) could be considered as first order [R3] (8, 15).

[R3] S(IV)+B3BOA∗→SO42−.

Despite the oxidative SO_2_ uptake to potentially acidify BBOA, prolonged illumination did not significantly reduce sulfate formation (Fig. 2 *A* and *B*).

The sulfate formation rate in BBOA can be given by

[1] d[SO42−]dt=kB3BOA∗,S(IV)[B3BOA*]×(1+Ka1[H+]+Ka1Ka2[H+]2)HSO2PSO2,

where K_a1_ and K_a2_ are the thermodynamic dissociation constants of H_2_SO_3_. H_SO2_ and P_SO2_ represent Henry’s law constant and partial pressure of SO_2_, respectively. The equilibrium constants are summarized in *SI Appendix*, Table S1.

### The Significant Role of Interfacial Reactions.

**Fig. 3.**
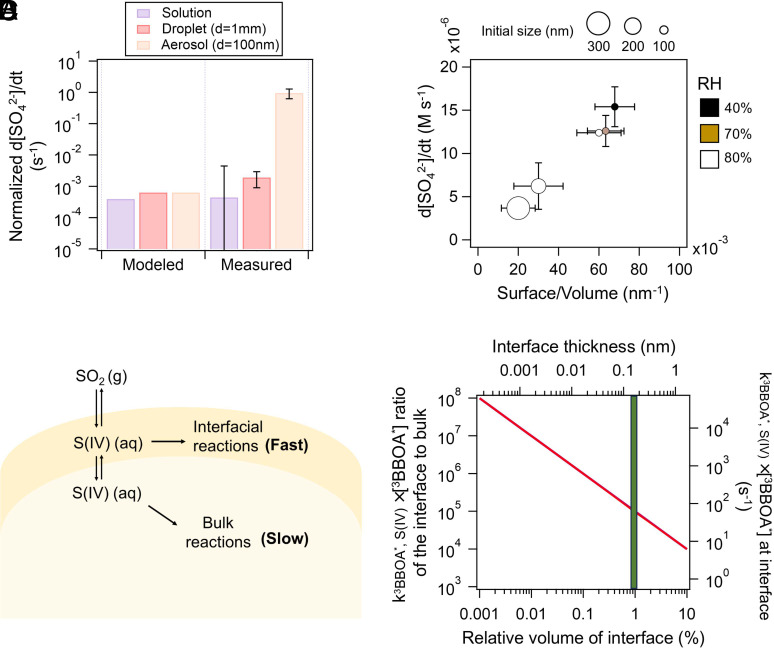
(*A*) The modeled and measured sulfate formation rate in bulk solution (purple), deposited droplets (red), and aerosol particles (pink), normalized by the concentration of S(IV). The sulfate formation rate in bulk solution was calculated up to 10% decay of S(IV), and the initial S(IV) concentration of 10 mM was used for normalization. The aerosol and deposited droplet experiments were conducted at 80% RH and 40 ppb SO_2_; (*B*) The sulfate formation rate as a function of the surface-to-volume ratio of the particles. The color and the symbol size show the RH and initial size of the BBOA particles, respectively. The equilibrated sizes at 70% and 40% RH are estimated by the size growth factor measured by H-TDMA; (*C*) The illustration for the distinct S(IV) oxidation kinetic between the interface and bulk phase of the BBOA particles; (*D*) The k_^3^BBOA^∗^, S(IV)_ × [^3^BBOA^*^] ratio of the interface to the bulk phase of the BBOA particles (*Left* axis) as a function of relative volume of the interface for a 100 nm BBOA particle (*Bottom* axis). The corresponding interfacial thickness and k_^3^BBOA^∗^, S(IV)_ × [^3^BBOA^*^] were shown on the *Top* and *Right* axes, respectively. The green column shows the typical thickness of the water monolayer. The BBOA particles were assumed spherical.

We first model the sulfate formation kinetics using Eq. **1**, based on data from diluted BBOA solutions mixed with S(IV) [BBOA to water mass ratio (BWR) of 10^−3^]. To compare results from solution and particle, we normalized the sulfate formation rate by the S(IV) concentration, prescribed in diluted solution and calculated by gas-particle partitioning, respectively. The pH of solution was adjusted to 4 to facilitate comparison. Fig. 3*A* shows that the normalized sulfate formation rate from modeling closely aligns with experimental measurements in the BBOA solution. This also suggested that the ^3^BBOA^*^ quenching by S(IV) measured via TA represents the reaction well. However, a stark discrepancy arises with BBOA particles, where the modeled normalized sulfate formation rate is three orders of magnitudes lower than the measured rate. This divergence indicates potential underpredictions of the k_^3^BBOA^∗^, S(IV)_ × [^3^BBOA^*^] in aerosols.

As the BWR increases, both the formation rate of ^3^BBOA^*^ and sink rate by organic matters (OM) increase, resulting in modeled [^3^BBOA^*^] reaching a plateau (*SI Appendix*, Fig. S5). Despite the comparable modeled [^3^BBOA^*^] between solution at BWR of 10^−3^ and aerosol (e.g., BWR of 1), aerosol particles have a significantly higher surface-to-volume ratio (S/V) than bulk solutions. Our modeling based on Eq. **1** assumes identical environment for both the interface and the bulk of the particles. However, in reality, molecular behavior at the interface could differ significantly from that in bulk solutions (51). For example, the interfacial semisolvation reduces solvent rearrangement energy (52) and stabilizes excited state molecules (53), thereby increasing the reaction rate constant. In addition, photosensitizers and ^3^C^*^s can be concentrated at the interface in the presence of trace amount of surfactant molecules (e.g., a monolayer), facilitating interfacial reactions (54–57).

Recent work by Wang et al. (40) observed accelerated S(IV) oxidation in illuminated humic acid microdroplets that generate reactive triplets and OH•. While the electron localization fields for the photosensitizers are mostly identical between the air–water interface and the bulk, the interface stabilizes the radicals generated by electron transfer. This phenomenon is also found in our molecular dynamic simulations with 3,4-dimethoxy benzaldehyde (DMB, a known photosensitizer in BBOA), which could support a higher k_^3^BBOA^∗^, S(IV)_ at the interface than bulk due to the reduced energy of the radical product (i.e., DMB^•−^) (*SI Appendix*, Figs. S6 and S7 and Text S3).

Using a flow cell, we exposed large deposited droplets (approximately 1 mm in diameter) to the same SO_2_ concentration, relative humidity (RH), and light intensity as we did for submicron aerosols in the AFT. Since the large droplets were fully equilibrated at 80% RH, their solute concentrations were expected to be the same as that of the submicron particles, with the only difference being the S/V. The sulfate formation rate in large BBOA droplets was slightly higher than that in diluted solutions but significantly lower than in submicron particles, further corroborating the importance of interfacial reactions (Fig. 3*A*). Additionally, an increase in sulfate formation rates was observed with higher S/V of submicron particles (Fig. 3*B*). This dependence holds even under two orders of magnitude higher viscosity at RHs (7, 58). Overall, the faster photosensitized sulfate formation in submicron aerosol particles compared to bulk solutions is attributed to prominent interfacial reactions rather than differences in pH or solute concentration.

We utilized a two-layer model comprising an interface layer and a bulk layer to illustrate the potential extent of acceleration for interfacial reactions over those in the bulk, as depicted in Fig. 3*C*. We assumed a fast equilibrium of S(IV) at the interface and the bulk, alongside a three times higher SO_2_ concentration at the air–water interface than that in the bulk predicted by Henry’s law (59). The normalized sulfate formation rate can be expressed as

[2] d[SO42−]dt[S(IV)]=(kB3BOA∗,S(IV),i×[B3BOA*]i×RVi×3)+(kB3BOA∗,S(IV),b×[B3BOA∗]b×RVb).

The subscripts i and b represent interface and bulk. RV denotes relative volume. Using the same k_^3^BBOA^∗^, S(IV), b_ and [^3^BBOA^*^]_b_ of the aerosol as that derived from solution (i.e., 5 × 10^8^ M^−1^ s^−1^ and 1.25 × 10^−12^ M), the k_^3^BBOA^∗^, S(IV), i_ ×[^3^BBOA^*^]_i_ across different RV_i_ in the BBOA particle can be obtained from the Eq. **2** (Fig. 3*D*).

Unique photochemistry has been reported to occur within a monolayer at the interface (51, 56, 57). Assuming a typical interfacial water monolayer thickness (L) of 0.15 nm (51), the RV_i_ is roughly 1% for 100 nm particles. Under these conditions, k_^3^BBOA^∗^, S(IV), i_ × [^3^BBOA^*^]_i_ reaches 10^2^ s^−1^, which is five orders of magnitude higher than that for the bulk (Fig. 3*D*). While it is not possible to precisely quantify the size of the interfacial reaction “zone”, increased thickness of the interfacial layer would lead to lower k_^3^BBOA^∗^, S(IV), i_ × [^3^BBOA^*^]_i_.

The mass accommodation coefficient (k_MT_) defines the fraction of collisions with a surface that lead to uptake of gas molecules to the surface, expressed as:

[3] $$k_{\text{MT}}=\left[\frac{R_{p}^{2}}{3D_{g}}+\frac{4R_{p}}{3\alpha v}\right]-1,$$

where R_p_, D_g_, α, and v are the radius of the particle, gas-phase molecular diffusion coefficient, mass accommodation coefficient, and mean molecular speed of SO_2_, respectively. Taking a typical α of 0.11 for SO_2_ (16), k_MT_ would be approximately 5 × 10^8^ s^−1^, which is much higher than the interfacial pseudo-first-order reaction rate constant with ^3^BBOA^*^ (k_^3^BBOA^∗^, S(IV), i_ × [^3^BBOA^*^]_i_ = 10^2^ s^−1^). This supported the fast equilibrium of S(IV) at the interface without mass-transfer limitation.

Taking the same diffusion coefficient (D) for S(IV) as that reported for ozone in the hydrophilic phase of BBOA particles (58), the characteristic time for S(IV) diffusion crossing interface ($\tau_{\mathrm{diff}}\;=\;\frac{L^{2}}{D}$) is 10^−9^ and 10^−11^ s at 40% and 80% RH, much shorter than the characteristic time for its interfacial reaction with ^3^BBOA^*^ (10^−2^ s,τreact=1kB3BOA∗,S(IV),i×[B3BOA∗]i). This implies that S(IV) is not confined to the interface. However, the interfacial reaction accounts for more than 99% of S(IV) oxidation in the BBOA. It should be noted that the rate constant for reactant diffusion and collision to form a precursor complex is typically 10^9^ M^−1^ s^−1^ in aqueous solutions (60). If this represents the upper limit for k_^3^BBOA^∗^, S(IV), i_, then the [^3^BBOA^*^]_i_ would reach 10^−7^ M, which was underestimated by five orders of magnitude using the extrapolated values from bulk solution measurements.

On the other hand, we observed that the decay of levoglucosan, a compound reported with no surface propensity and likely located in the BBOA bulk (61), is similar to the modeled results using the quenching rate constants and the predicted [^3^BBOA^*^] from diluted solution (*SI Appendix*, Fig. S8). This suggested that the k_^3^BBOA^∗^_ × [^3^BBOA^*^] may not be elevated for bulk phase reactions in the BBOA particles, assuming that the production of levoglucosan via back electron transfer to the oxidized levoglucosan is insignificant. Overall, an averaged enhancement factor (EF) of three orders of magnitude from the solution value can represent the k_^3^BBOA^∗^, S(IV)_ × [^3^BBOA^*^] in BBOA particles for multiphase reactions.

### The Photochemical Reactivity of the BBOA Photosensitizers.

Understanding how different photosensitizers in BBOA promote multiphase reactions is of great significance, given the expected variability of photosensitizer speciation in the ambient atmosphere. To simplify, we have treated the ^3^C^*^ species in BBOA as a single entity in the above analysis (i.e., ^3^BBOA^*^, Eq. **1**). Here, we consider the sulfate formation kinetics driven by *N* different ^3^C* species:

[4] d[SO42−]dt=∑i=1NkC3i∗,S(IV)[C3i∗]×(1+Ka1[H+]+Ka1Ka2[H+]2)HSO2PSO2.

At steady state, the concentration of each ^3^C^*^ species can be derived from its formation rate [the product of the light absorption rate (R_abs_) and the intersystem crossing quantum yields (Ф_ISC_)] over the total sinking rates by OM and S(IV):

[5] [ 3Ci∗]=Rabs,PSi×ФISC,PSik 3Ci∗,OM[OM]+k 3Ci∗,S(IV)[S(IV)].

**Fig. 4.**
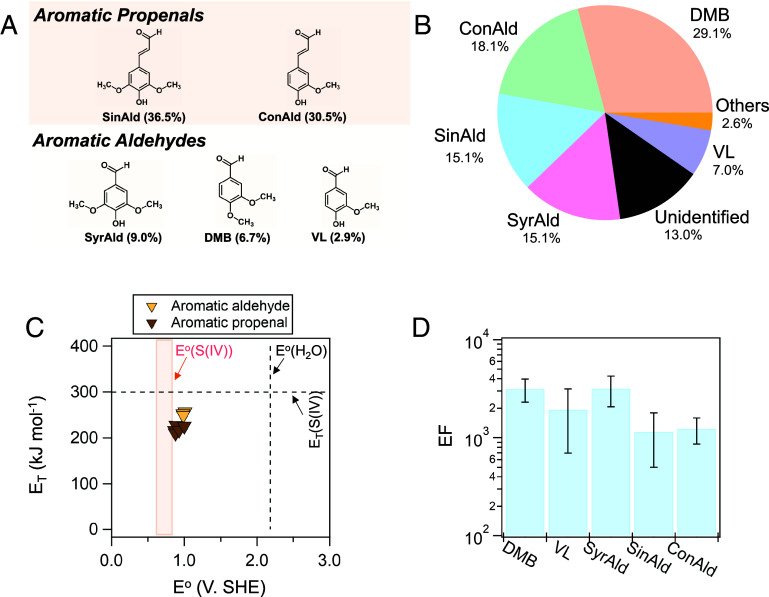
(*A*) The structures of the major chromophores identified. The numbers in the blanket are the percentage contribution to the total PDA absorption; (*B*) The contribution of sulfate formation by different types of photosensitizers according to the enhanced sulfate formation by doubling the concentrations of the corresponding photosensitizer; (*C*) The map of energy and one-electron reduction potential for different ^3^C^*^ of identified photosensitizers in BBOA. V. SHE indicates the volts relative to the standard hydrogen electrode. The data are summarized in *SI Appendix*, Table S2; (*D*) The EF of normalized sulfate formation in aerosol particles over bulk solution. Before EF calculation, the sulfate formation rates were normalized by S(IV) concentrations in surface and bulk, respectively. Both aerosol particles and bulk solutions contain 1 mM photosensitizers and around 3 M sodium bimalonate/sodium malonate=1:1 buffer.

We identify the potential photosensitizers and characterize their light absorption in our BBOA sample. The two most prevalent chromophores at 300 to 400 nm were Sinapaldehyde (SinAld) and Coniferyl aldehyde (ConAld). Each of them accounts for more than 30% of the PDA absorption. Other known photosensitizers including DMB, syringaldehyde (SyrAld), and vanillin (VL) contributed moderately (2.9 to 9%) to PDA absorption. These major chromophores can be primarily categorized into aromatic propenals and aromatic aldehydes, with structures shown in Fig. 4*A*. The former compounds likely originated from lignin degradation and the subsequent oxidation forms the latter compounds (*SI Appendix*, Fig. S9). Their conjugated C = O moieties, not only enhance the light absorption by electron withdrawal from the aromatic ring (62) but also facilitate intersystem crossing through coupled n and π orbitals, making them potential photosensitizers (63).

By doubling the concentrations of each major chromophore and measuring the enhanced sulfate formation rate, we estimated their contributions to the sulfate formation, assuming a proportional increase in sulfate formation. In the BBOA collected from the AFT without illumination, the ratio of photosensitizers to K^+^ [used as an internal standard due to its stability toward photochemical reactions (23, 64)] decreased by less than 8% compared to the original solution for atomization. This indicates minimal evaporation of photosensitizers, consistent with their relatively high Log K_wa_ and Log K_oa_ (*SI Appendix*, Table S2). Fig. 4*B* shows that these identified chromophores accounted for most of the sulfate formation under light, with contributions descending in order: DMB (29%) > ConAld (18%) > SinAld (15%) = SyrAld (15%) > VL (7%). Minor contributors such as Ferulic acid, Vanillylidene acetone, and 4-methoxy-cinnamaldehyde collectively accounted for 2% of the enhanced sulfate formation and are denoted as “others.”

Despite the lower contribution to PDA intensity than SinAld and ConAld, DMB was the greatest contributor to sulfate formation, which implies different reactivities and ISC quantum yield among ^3^C^*^s. We calculated the one-electron reduction potential (E^o^) and triplet energy (E_T_) of the ^3^C^*^ of these photosensitizers (*SI Appendix*, Text S4), which can reflect their reactivity via electron transfer and energy transfer, respectively, as displayed in Fig. 4*C*. It should be noted that we focused on the unprotonated ^3^C^*^ for the E_T_ and E^o^ map in Fig. 4*C* and that the protonation at low pH can increase the E^o^ of the ^3^C^*^ to promote reactions (*SI Appendix*, Table S2) (11, 65). The aromatic propenals and aromatic aldehydes exhibited similar E^o^s of around 1 V. SHE, higher than the one-electron oxidation potential of S(IV) [0.63 to 0.84 V. SHE (66, 67)], indicating that electron transfer from S(IV) to ^3^BBOA^*^ is thermodynamically favorable. The logarithm of the second-order quenching rate constants of ^3^C^*^ by S(IV) does not increase with the difference between the one-electron reduction potential of ^3^C^*^ and the oxidation potential of S(IV), suggesting that a diffusion-control regime has been approached (*SI Appendix*, Fig. S10). Furthermore, the DMPO-SO_3_ peaks detected in the illuminated BBOA and NaHSO_3_ mixture under N_2_ conditions support the electron transfer from S(IV) to ^3^BBOA^*^ that yields SO_3_**^•−^** (*SI Appendix*, Fig. S11). The significant decay of TEMPO intensity in the EPR spectra of the illuminated BB and NaHSO_3_ mixture also serves as evidence for the electron-transfer reactions (68). The E^o^ of ^3^C^*^s in BBOA are mostly below the one-electron oxidation potential of water [e.g., 2.18 V. SHE (69)], rendering the electron transfer from water or OH^−^ to form OH• unfavorable, consistent with the minimal OH• production. DMPO-OH was detected in the illuminated mixed solution of benzoquinone (BQ, E^o^ = 2.42 V.SHE) and DMPO, but not in BBOA, DMB, and VL solutions at the same mass concentration (*SI Appendix*, Fig. S12). This might explain the recent observation by Liu-Kang et al. (70), which suggested a minor role of OH• in secondary BrC formation in illuminated BBOA. For energy transfer reactions, the E_T_s of aromatic aldehydes are slightly higher than that of aromatic propenals, but still below the required value to excite S(IV) (e.g., 300 kJ mol^−1^) (71). Hence, sulfate formation from these photosensitizers is likely due to electron transfer rather than energy transfer reactions. Also, the similar E_T_ and E^o^ suggested that the ^3^C^*^ reactivities of aromatic propenals and aromatic aldehydes toward S(IV), and maybe OM, are likely close.

The Ф_ISC_ of ^3^C^*^, another determinant of [^3^C^*^] besides the light absorption rate, plays a key role in their contributions to sulfate formation. Assuming the same ^3^C^*^ reactivity, the enhanced sulfate formation rate after doubling the concentration of the photosensitizers is proportional to the R_abs_ and Ф_ISC_, simplified in Eq. **6**:

[6] $$\Delta \frac{d[{\text{SO}}_{4}^{2-}]}{\text{dt}}\propto R_{\text{abs}}\times {Ф}_{\text{ISC}}.$$

Taking the PDA absorption intensity as R_abs_ and the reported Ф_ISC_ for VL (0.21) and DMB (0.38) (8), we estimated the Ф_ISC_ for SinAld (0.04), ConAld (0.05), and SyrAld (0.14) based on sulfate formation results. Aromatic aldehydes exhibited generally higher Ф_ISC_ (24 ± 12%), making them more effective photosensitizers than aromatic propenals (Ф_ISC_ = 5 ± 1%). Despite the low Ф_ISC,_ aromatic propenals still contribute to the sulfate formation, benefiting from their around 2 to 3 folds higher molar absorptivity than aromatic aldehydes (11). The larger steric effects by vinyl groups in aromatic propenals may hinder electron transfer reactions, potentially leading to an underestimation of their Ф_ISC_.

To conclude, aromatic aldehydes possess higher ISC quantum yields and lead to higher [^3^C^*^] than aromatic propenals to drive multiphase oxidation. The relative abundance of aromatic aldehydes to aromatic propenals can vary across different types of BBOA, though our results align with those reported by the literature, which showed that the light absorption by aromatic propenals prevails over aromatic aldehydes (72, 73). More importantly, we found that for those identified major photosensitizers, the average EF of sulfate formation in aerosol particles over the bulk solution was (2.1 ± 0.3) ×10^3^ (Fig. 4*D*), which is similar to the EF for BBOA (approximately 3 × 10^3^).

### Sulfate Formation by ^3^BBOA^*^ Chemistry in Wildfire.

The transport of wildfire smoke in the atmosphere exhibits significant spatial and temporal complexities. Although it is currently not feasible to parameterize photosensitization reactions in BBOA in global models, the sulfate formation kinetics and behaviors of photosensitizers detailed in this study highlight the potential impact of multiphase BBOA photochemistry on air quality in wildfire-prone regions during fire seasons (74).

For comparative analysis, we calculated the sulfate formation through H_2_O_2_ oxidation, which has been reported to dominate global sulfate production (75), using local concentrations of SO_2_, H_2_O_2_, pH, and aerosol liquid water. We considered BBOA concentrations of 10 and 40 µg m^−3^, representing moderate and severe fire conditions, respectively. Details of these calculations are provided in *SI Appendix*, Table S3 and Text S5. We focused on the direct reactions between ^3^BBOA^*^ and S(IV) for sulfate production. It is noteworthy that the presence of O_2_ will greatly complicate the chemistry. Specifically, ^3^BBOA^*^ will react with the dissolved O_2_ via energy transfer to form ^1^O_2_^*^ (76, 77). The HBBOA, formed via hydrogen abstraction from OM by ^3^BBOA^*^, can rapidly react with O_2_ to generate HO_2_ (78), which subsequently leads to H_2_O_2_ and OH• (79). The ^1^O_2_^*^, HO_2_/O_2_^•−^, H_2_O_2_, and OH• are collectively known as “secondary oxidants,” which can also contribute to the S(IV) oxidation (25). Sulfate formation in BBOA in air is around double of that in N_2_ (*SI Appendix*, Fig. S13). While resolving the concentrations of each secondary oxidant is beyond the focus of this work, we did not expect different [^3^C^*^] between air and N_2_ conditions since OM will be a predominant sink for ^3^C^*^ in both cases, due to its much higher concentration (>30 M C) than dissolved oxygen (10^−4^ M) in BBOA particles. Moreover, we do not explicitly consider the emission of primary precursors (e.g., SO_2_) even for the severe fire scenario, which may also lead to underestimations.

**Fig. 5.**
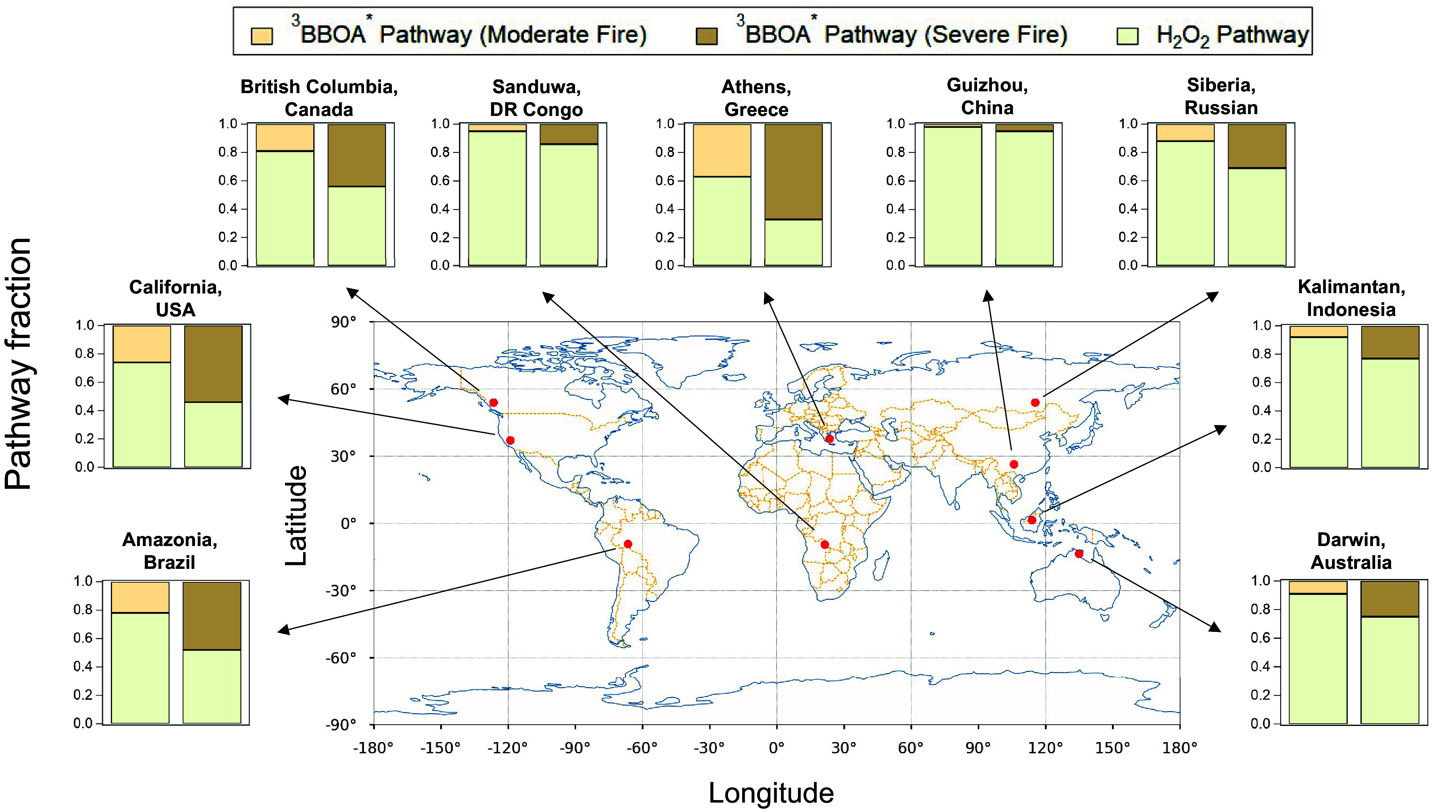
Sulfate formation in wildfire-prone regions. The histograms show the fractional contributions of the ^3^BBOA^*^ and H_2_O_2_ oxidation pathways to the sulfate formation under different fire severity.

According to the TUV model (*SI Appendix*, Text S5), an increase in BBOA loading from 10 to 40 µg m^−3^ attenuates the photon flux by 10 to 30%, which reduces the formation of ^3^BBOA^*^ and partially offsets the increased sulfate formation (Fig. 5). Nonetheless, in regions like British Columbia, California, Amazonia, and Athens, the fractional contribution of ^3^BBOA^*^ to sulfate formation increases significantly with fire severity to about 0.5 to 0.7, surpassing that by H_2_O_2_. In contrast, in Asian and African regions such as Guizhou and Leopoldville, the increase is more moderate at 0.04 and 0.09, respectively. It is important to note that we calculated the UV flux at the ground level to provide a conservative estimate of photosensitized sulfate formation, especially for severe fire scenarios.

The increase in sulfate formation rate by ^3^BBOA^*^ upon rising fire severity shows a negative correlation with aerosol pH but there is no clear correlation with SO_2_ or H_2_O_2_ concentrations (*SI Appendix*, Fig. S14). At pH above 2, where the increased SO_2_ solubility compensates for the reduced reaction rate constant, the sulfate formation rate by H_2_O_2_ oxidation is largely pH-independent (*SI Appendix*, Fig. S15) (16). At pH below 2, which applies to most cases in Fig. 5, the H_2_O_2_ sulfate formation rate decreases significantly due to a shift from HSO_3_^−^ to less reactive H_2_SO_3_ as the dominant S(IV) species. On the other hand, the ^3^BBOA^*^ sulfate formation is less sensitive to pH due to the comparable reactivity of ^3^BBOA^*^ toward different S(IV) species. Therefore, enhanced sulfate formation driven by ^3^BBOA^*^ in wildfires could be particularly significant in regions with lower aerosol acidity.

## Discussion

Our results demonstrated that BBOA exhibited prominent photochemical reactivity, which can effectively drive multiphase reactions such as SO_2_ oxidation to sulfate. We further show that using concentrations and rate constants on photosensitization from bulk experiments can underestimate multiphase sulfate formation in aerosols by three orders of magnitude. Besides aromatic photosensitizers in BBOA, 2-imidazole carboxaldehyde (2-IC), a common atmospheric photosensitizer used in laboratory studies, gave comparable EF (*SI Appendix*, Fig. S16). While the direct determination of reaction rate constants and ^3^C^*^ concentrations remains challenging, an EF of three orders of magnitude may be used to estimate the k_^3^C^∗^_ × [^3^C^*^] in BBOA particles for multiphase reactions, based on parameters derived from the bulk. For example, extrapolation from the solution data yields [^3^C^*^] of 10^−13^ M in ambient particles (*SI Appendix*, Fig. S17). Using this estimate and the reaction rate constant for ambient extract (bulk solution) (15), the sulfate formation rate of 10^−4^-10^−2^ µg m^−3^ h^−1^ can be obtained at pH of 4 to 6, as typical scenarios of Eastern Asian haze. After applying the EF, the sulfate formation rate in particles is estimated as 10^−1^ to 10 µg m^−3^ h^−1^, which aligns with the “missing” sulfate formation rate of 0.3 to 5 µg m^−3^ h^−1^ between modeling and field measurements.

The diverse speciation and reactivities of photosensitizers in BBOA have greatly complicated parameterization. As a step forward, we identified the potential photosensitizers in BBOA, profiled their E_T_ and E^o^, and correlated with their abilities in oxidizing S(IV) to sulfate. The results suggested that the tested BBOA photosensitizers may possess overall similar reactivities and varied Ф_ISC_ by substitution. To our best knowledge, there is a limited number of identified photosensitizers in the atmosphere. Aromatic aldehydes, such as DMB, are commonly used as model BB photosensitizers in laboratory investigations, and our results support their validity (8).

With the photochemical properties of ^3^C^*^s, it could be feasible to parameterize photosensitized reactions in atmospheric waters containing a complex mixture of photosensitizers. Specifically, the reaction rate of a substrate (S) with ^3^C^*^s can be expressed as Eq. **7**, by combining Eqs. **4** and **5**:

[7] d[S]dt=∑i=1Nk 3Ci∗,S×[S]      ×Rabs×ФISCk 3Ci∗,S[S]+k 3Ci∗,OM[OM]+k 3Ci∗,O2[O2].

k_^3^C^∗^i, S_, k_^3^C^∗^i, OM_, and k^3^C^∗^i, O_2_ denote the second-order quenching rate constant of ^3^C^*^ by the substrate, OM (other than the substrate), and O_2_, which could be measured and estimated from the E^o^ and E_T_ (e.g., using Rehm–Weller equation). In BBOA particles where the reactivities of ^3^C^*^s are potentially similar, and OM likely prevails over the other sinks, Eq. **7** can be simplified to Eq. **8**:

[8] d[S]dt=k 3C∗,S[S]k 3C∗,OM[OM]×∑i=1NRabs,i×ФISC,i.

R_abs_ can be derived from the concentration of the aromatic aldehydes and aromatic propenals and their molar absorptivity. With the corresponding Ф_ISC_, one can predict the substrate oxidation by ^3^BBOA^*^ when the k_^3^C^∗^i, S_ and k_^3^C^∗^i, OM_ are available. An EF of 10^3^ is recommended for multiphase reactions in aerosol particles. It is worth mentioning that a combined approach using ultrafast spectroscopy and chemical probes has been reported as effective for quantifying the quantum yield of short-lived oxidants in real time (80).

In this study, we focus on the BBOA derived from water extracts, given that the BB plume directly from the furnace was not stable enough for kinetics and the organic solvent will interfere with probe experiments. It should be noted that the photosensitizers identified in the BBOA (water extract) still have much lower water solubilities (e.g., 10^−2^ M) than the atmospheric organics considered highly water-soluble, such as glyoxal (~2 M). GC×GC ToF MS analysis of the hexane extracts targeting the least water-soluble compounds showed that single-ring aromatics, led by aromatic propenals and aromatic aldehydes, are still predominant (*SI Appendix*, Fig. S18). This implies that the majority of photosensitizers in BB particles are likely included in water extracts. However, it should be noted some compounds with even lower water solubilities (e.g., <10^−3^ M) than single-ring aromatics, such as polyaromatic hydrocarbons may still be less recovered by water extraction (*SI Appendix*, Table S2) (72). In aerosol particles with limited liquid water, these poorly water-soluble compounds with high Log K_oa_ values may form a hydrophobic shell of the particles, leaving the more soluble counterparts in the hydrophilic core (*SI Appendix*, Fig. S19) (58). These less soluble compounds generally exhibit stronger light-absorption capacities due to their planar and conjugated structures, making them potentially good photosensitizer candidates (81). Moreover, aromatic aldehydes and aromatic propenals can partition into the hydrophobic shell owing to their high Log K_oa_ (around 10) and drive multiphase reactions after being excited to their triplet states. Though kinetic analysis was not possible, we found the enhanced sulfate formation in BB particles (directly from the furnace without undergoing extraction) over that in BBOA (water extract) under light, indicated by the mass ratio of SO_4_^2−^ to K^+^ (*SI Appendix*, Fig. S20).

The aging of BBOA during atmospheric transportation has been reported to show initial enhancements in light absorption, followed by photobleaching at slower rates (6, 70, 82). The typical BrC lifetime at ground levels is about 1 d or less (7, 83). However, when BBOA is elevated to higher altitudes, such as 5 km, through deep convection processes, the lower temperatures significantly increase its viscosity (84, 85). Such elevated viscosity could extend the lifetime of primary photosensitizers against oxidative bleaching by gaseous oxidants by 2 to 5 orders of magnitude, compared to conditions at 1 km altitude (7). Though increased viscosity hinders diffusion of gaseous precursors, the multiphase reactions triggered by interfacial photosensitization may still be effective. The rapid conversion of BBOA to oxidized organic aerosol (OOA) is well documented (86). However, OOA from biomass burning has also been identified as an important contributor to atmospheric ^3^C^*^(87). Despite the potentially limited diffusion of gas phase oxidants, photosensitization could serve as a source of “in-particle” oxidants to trigger particle aging and changes in physicochemical properties.

Beyond S(IV), the reactions between ^3^BBOA^*^ and other atmospheric precursors, such as volatile organic compounds, warrant further quantitative investigation (88). The E_T_ and E^o^ of ^3^BBOA^*^ provide valuable references for inferring and parameterizing its reactivities toward various atmospheric precursors. Subsequent studies are also encouraged to explore how these photosensitization reactions depend on the combustion condition, environmental factors, and atmospheric aging of BBOA, to better understand their impacts on air quality and climate. Approaches for identifying the potential photosensitizers in diverse and complex particulate matrix will be invaluable.

## Materials and Methods

### Preparation of BBOA.

Biomass-burning (BB) particles were generated by smoldering untreated pine wood sourced from a Canadian hardware store. The apparatus for generating BB particles is shown in *SI Appendix*, Fig. S21. In brief, untreated pine wood was cut into strips (~20 × 2 × 2 mm) and positioned lengthwise inside a quartz flow tube furnace (inner diameter 36 mm, outer diameter 40 mm, length 1,400 mm). Zero air (Peak Scientific) was introduced through the tube at a flow rate of 2 L min^−1^ using a mass flow controller (Alicat). The heating temperature within the tube was calibrated and maintained at 400 °C, in the range of natural biomass burning (e.g., 300 to 800 °C) (6, 58). Aethalometer (USA) measurement shows that the black carbon concentration drops back to the baseline level after 1 min of heating, indicating a transition from the flaming phase to the smoldering phase. Therefore, the samplings were performed approximately two min after the temperature reached 400 °C. After dilution using a two-stage jet flow dilutor (Dekati), the BB particles from the smoldering phase were collected onto a 47 mm quartz filter (Pall) loaded on a stainless-steel filter holder. Prior to particle collections, the quartz filters were prebaked at 550 °C for 24 h to remove the organic residuals. BBOA was obtained by extracting the collected BB particles using ultrapure water, under sonication, followed by filtration through 0.22 µm Teflon filters to remove the insoluble components. The chemical composition of chromophores in the BBOA was characterized by high-performance liquid chromatography with a photodiode array and high-resolution orbitrap mass spectroscopy (HPLC-PDA-HRMS) (*SI Appendix*, Text S6). Chemical standards were used to identify the main chromophores. The light absorption pattern of the BBOA was determined by a UV-Vis spectrophotometer (Agilent) (*SI Appendix*, Fig. S22). Nonpolar components were extracted with hexane and characterized by two-dimensional gas chromatography coupled with time-of-flight mass spectrometry (GC×GC ToF MS), providing broad profiling of the particle constituents (*SI Appendix*, Text S7). Additional analyses, including Inductively Coupled Plasma Mass Spectrometry (ICP-MS, ThermoFisher), ion chromatogram (IC, ThermoFisher), and total organic carbon analyzer (TOC, Agilent) were used to quantify the ionic species and total organic carbon. All solvents used in the study are LC-MS grade from VWR. In addition, the BB particles were also deposited onto a glass slide coated with superhydrophobic and oleophobic material, using a PM_2.5_ impactor (TSI). An optical photothermal infrared microscope (O-PTIR, Photothermal Spectroscopy Corp.) was used to characterize the morphology and spatial distribution of the chemical components within the BB particles (*SI Appendix*, Text S8). A Hygroscopicity Tandem Differential Mobility Analyzer (BMI) was used to determine the size growth factor of the BBOA at different RHs, as detailed in our previous work (23) (*SI Appendix*, Fig. S23).

### Flow Tube Experiments.

A stream of BBOA particles was generated using a constant output atomizer (TSI) and a flow of 0.1 L min^−1^ was first prehumified to reach equilibrium at 80% RH, then passed through a charcoal denuder to remove the potential volatile organic compounds (VOC). Subsequently, a differential mobility analyzer (DMA) selected the size of the BBOA particles, which were then introduced to an aerosol flow tube (AFT, 10 cm internal diameter, 120 cm length) made of quartz (*SI Appendix*, Fig. S24). The Reynold number in the AFT is around 11. A flow of SO_2_ carried by mixed dry-wet N_2_ (1.4 L min^−1^) was also introduced to the OFR to interact with the BBOA particles. The residence time of the BBOA particles in the OFR is approximately 380 s.

UVA light tubes (with a continuous emission spectrum over 300 to 420 nm) were evenly mounted along the length of the AFT on its outside (*SI Appendix*, Fig. S22). The photon flux to the AFT is comparable to the actinic flux at noon in the summer of British Columbia, Canada. The details for the determination of photon flux are available in *SI Appendix*, Text S9. At the exhaust of the AFT, an M170 digital sensor (Vaisala) was used to monitor the temperature and RH. The temperature for all experiments was around 293 K. The SO_2_ concentration and particle number concentrations were measured by an SO_2_ analyzer (Thermo Fisher) and a condensation particle counter (CPC, BMI). A noticeable increase in the particle number concentration was not observed when the light turned on, therefore, the new particle formation due to photochemistry was not considered.

We used 47 mm Teflon filters loaded in an in-line stainless steel filter holder to collect the aged particles (by SO_2_) at the exhaust. After that, the filters were extracted by ultrapure water and the sulfate moles in the aqueous extract were measured by IC. The sulfate concentration in the BBOA particles (M) can be quantified as

[9] $$\left[{\text{SO}}_{4}^{2-}\right]\left(M\right)=\frac{\mathrm{mole}({\text{SO}}_{4}^{2-})}{\frac{m_{p}}{\rho_{\text{BBOA}}}},$$

where m_p_ is the mass loading of the particles measured by a calibrated microbalance (*SI Appendix*, Fig. S25 and Text S10). ρ is the density of BBOA assumed as 1.3 g cm^−3^ (89). A movable stainless-steel injector tube (inserted axially down the center of the flow tube) allowed variation in light exposure time, thus we can quantify the sulfate formed in BBOA particles as a function of illumination time and calculate the sulfate formation rate (*SI Appendix*, Table S4). We assumed no effects on particle size and density by considering the maximum abundance of the formed sulfate with the water it absorbs. To compare the sulfate formation initiated by individual photosensitizers between bulk solution and aerosol particles, we mixed the photosensitizers with organic buffer (sodium malonate/sodium bimalonate = 1:1), which provides aerosol liquid water to minimize the evaporation of photosensitizers.

Sulfate formation rates in particles and droplets with different surface-to-volume ratios (S/V) provide insight into the significance of interfacial reactions. To investigate this, we applied different voltages to the DMA for submicron particles of varying sizes (100 to 300 nm). We also conducted SO_2_ uptake experiments on large deposited BBOA droplets (diameter of approximately 1 mm after equilibrium) using a flow cell, as detailed in our previous works (90–92). In brief, 1 μL BBOA droplets were deposited onto hydrophobic substrates and equilibrated at 80% RH inside the flow cell. An optical microscope (Leica) equipped with a microruler was used to monitor the size change, ensuring equilibrium. Once equilibrium was achieved, pre-equilibrated SO_2_ steam was introduced into the flow cell, and the droplets were illuminated from the top–down using UVA light through the cell’s quartz window. The droplets were sampled at different time points, extracted, and the sulfate concentration was determined using IC for kinetic analysis.

### Determination of the ^3^BBOA^*^ Concentration.

Syringol (SYR) was used as an internal chemical probe to determine the production rate of the ^3^C^*^ of BBOA (i.e., ^3^BBOA^*^) in solution upon illumination using a quartz cuvette reactor. The solution temperature was maintained at around 293 K using a temperature-controlled plate and cycling water bath. UVA light tubes were positioned above the reactor, simulating the photon flux conditions similar to those in the AFT.

The rate constant for the SYR loss (k, s^−1^) under light illumination was determined. To obtain the pseudo-first-order SYR decay rate constant due to ^3^BBOA^*^ (k_SYR,^3^BBOA^∗^_[^3^BBOA^*^], s^−1^), we subtracted k by the SYR direct photodegradation rate constant under UVA illumination (j, s^−1^) and pseudo-first-order oxidation rate constants by OH• (k_SYR,OH•_[OH•], s^−1^) and ^1^O_2_^*^ (k_SYR,^1^O_2_^∗^_[^1^O_2_^*^], s^−1^):

[10] kSYR,B3BOA∗[B3BOA∗]=k−(j+kSYR,OH•[OH•]+kSYR, 1O2∗[ 1O2∗]).

The steady-state concentrations of these oxidants were determined by separately spiking benzoic acid (BA) and furfural alcohol (FFA) into the BBOA solution and monitoring the formation of p-hydroxybenzoic acid (p-HBA) and FFA decay via HPLC-PDA (78). The light screening effects on the probes were corrected (33).

k_SYR,^3^BBOA^∗^_, k_SYR,OH•_, and k_SYR,^1^O_2_^∗^_ are the bimolecular rate constants of SYR reacting with ^3^BBOA^*^, OH•, and ^1^O_2_^*^, respectively. We assumed that ^3^BBOA^*^ shared the same second-order reaction rate constant as the ^3^DMB^*^ with SYR [3.9 × 10^9^ M^−1^ s^−1^ (93)] to calculate the steady-state [^3^BBOA^*^], based on Eq. **10**.

Although SYR has been reported to undergo dark reactions that form BrC, particularly in the presence of Cl salts (94), we considered this pathway to be minor as negligible SYR decay was observed in the dark (*SI Appendix*, Fig. S26). It is also important to note that SYR has a high triplet energy [330 to 340 kJ mol^−1^ (95, 96)]. However, our analysis revealed that the major ^3^C* species in BBOA exhibit E_T_ values below 300 kJ mol^−1^, making energy transfer from SYR to ^3^C^*^ unlikely. On the other hand, the one-electron oxidation potential of SYR [approximately 0.7 V.SHE (97)] is lower than the E^o^ of the ^3^C^*^ in BBOA. Therefore, we expect that the majority of ^3^C^*^ were effectively quenched by SYR. The SYR decay could be subjected to back-electron donation of some electron-rich BBOA components and lead to underestimation of [^3^BBOA^*^], known as inhibition. The inhibition factor was quantified and used to correct [^3^BBOA^*^], according to Ma et al. (33) and Ma et al. (32). Overall, we considered the limitation of using SYR minor in this study.

The [^3^BBOA^*^] in concentrated aerosols cannot be directly measured, therefore we estimated it according to the formation rate (F_T_) and sink rates, derived from bulk measurements at a series of [OM] (*SI Appendix*, Table S5). At the steady state, the formation rate of ^3^BBOA^*^ equals its removal rate by OM and O_2_:

[11] FT=jabsΦISCf[OM]=(kB3BOA∗,OM[OM]+kB3BOA∗,O2[O2])[B3BOA∗],

where j_abs_ is the light absorption rate constant, Ф_ISC_ is the intersystem crossing quantum yield, f is the fraction of photosensitizers in the BBOA mixture, and k_^3^BBOA^∗^, OM_ and k_^3^BBOA^∗^, O_2__ are the second-order quenching rate constants of ^3^BBOA^*^ by OM and O_2_, respectively.

At low concentrations of BBOA at which O_2_ is the dominant sink (*SI Appendix*, Fig. S5), F_T_ can be determined by Eq. **12**:

[12] FT=k​B3BOA∗,O2[O2][B3BOA∗].

It should be noted diluted solution ([BBOA]/[H_2_O] = 10^−5^) was used here to derive F_T_ specifically. For BBOA particles ([BBOA]/[H_2_O] = 1) discussed in the main text, O_2_ is a minor sink of ^3^BBOA^*^ compared to OM. k_^3^BBOA^∗^, O_2__ is determined as 1 × 10^9^ M^−1^ s^−1^ based on transient absorption spectroscopy (will be introduced later), consistent with reported values for various aromatic photosensitizers (8). [O_2_] is the concentration of dissolved oxygen in the solution measured by a digital meter (Vio).

Rearrangement of Eq. **11** yields:

[13] [B3BOA∗]=(jabsΦISCfk​B3BOA∗,O2[O2])[OM]1+(k​B3BOA∗,OMk​B3BOA∗,O2[O2])[OM].

[^3^BBOA^*^] at a series of [OM] in bulk solution was used to fit Eq. **13** according to $y=\frac{a[\mathrm{OM}]}{1+b[\mathrm{OM}]}$. k_^3^BBOA^∗^, OM_ can be calculated according to b and k_^3^BBOA^∗^, O_2__.

Finally, the [^3^BBOA^*^] in aerosol particles, in the presence of SO_2_ in N_2_ can be estimated by

[14] [B3BOA∗]=FTk​B3BOA∗,OM[OM]+k​B3BOA∗,S(IV)[S(IV)].

### Transient Absorption Spectroscopy (TA).

A laser flash photolysis system coupled with an excitation laser at 310 nm was used to measure the ^3^BBOA^*^ quenching kinetics in the solution. A peristaltic pump was used to continuously supply the external BBOA solution to a quartz cuvette located inside the equipment, minimizing the effect of photobleaching on the kinetic measurements. For the ^3^BBOA^*^ quenching experiment by S(IV) and levoglucosan (used as a control experiment for the reactions in the bulk phase of the particle), the solution was purged with ultrapure N_2_ before the measurements to minimize ^3^BBOA^*^ quenching by O_2_.

^3^BBOA^*^ was generated by a laser pump followed by decay. The decay of ^3^BBOA^*^ was fitted with a single exponential term, to obtain the k_obs_. k_obs_ for absorption peaks at different wavelengths were averaged to yield the overall observed ^3^BBOA^*^ quenching rate constant (15):

[15] $$y=a+be^{-k_{\mathrm{obs}}t}.$$

The k_obs_ were measured at a range of quenchers such as [S(IV)]. According to the Stern–Volmer equation (Eq. **16**), the slope and intercept of the plot k_obs_ vs. [S(IV)] yield the ^3^BBOA^*^ quenching rate constant by S(IV) (k_^3^BBOA^∗^, S(IV)_) and the total natural deactivation rate of ^3^BBOA^*^ via self-quenching, phosphorescence, etc., (k_d_), respectively.

[16] −d[B3BOA∗]dt=(kd+kB3BOA∗,S(IV)[S(IV)])[B3BOA∗]=kobs[B3BOA∗].

Since the speciation of S(IV) is pH-dependent, we performed quenching experiments at pH = 1, 4, and 7 (by the addition of H_2_SO_4_ and NaOH), in which the SO_2_·H_2_O, HSO_3_^−^, and SO_3_^2−^ dominate (*SI Appendix*, Fig. S3), respectively. For the quenching kinetics between ^3^BBOA^*^ and O_2_, we purged the solution using mixed N_2_ and O_2_ flow to achieve different O_2_ content, and the dissolved oxygen content was monitored.

### Electro-Paramagnetic Resonance (EPR) Spectroscopy.

A continuous-wave EPR (CW-EPR) spectrometer (Bruker) coupled with a spin-trapping technique was used to measure the free radical formation in aqueous solutions under light. EPR peaks are generated by the absorption of microwave radiation by unpaired electrons in the presence of a magnetic field. The EPR peak intensity is directly proportional to the concentration of the radicals in the sample. We used a spin-trapping agent, 5,5-Dimethyl-1-pyrroline N-oxide (DMPO, Enzo), to convert the transient radicals such as OH to those with longer lifetimes such as DMPO-OH for measurements. The persistent 2,2,6,6-tetramethyl-1-piperidinyloxyl (TEMPO, Enzo) free radical was used to capture the dissolved electron during electron transfer from ^3^BBOA^*^. Specifically, the decay in TEMPO intensity indicates a reduction of TEMPO to the EPR-silent TEMPOH by dissolved electrons.

The spin-trap was spiked into the sample solution to make a final concentration of 0.1 M, and then 50 μL aliquot of the mixture was loaded into a 50 μL capillary tube and inserted in the resonator of the EPR spectrometer for temporal measurements over 90 min. An in situ UV irradiation system (Bruker) equipped with a 100 W Hg lamp was used with EPR. A safety shutter between the lamp and the resonator was used to control the start and stop of irradiation. The operating parameters for EPR measurements were as follows: a center field of 3,515.0 G, a sweep width of 100.0 G, a receiver gain of 25 dB, a modulation amplitude of 1.0 G, a scan number of 8, attenuation of 20 dB, a microwave power of 2 mW, a modulation frequency of 100 kHz, a microwave frequency of 9.86 GHz, and a conversion time and time constant of 5.12 ms. The Xenon software was used to calculate the peak area for the radical adducts.

## Supplementary Material

Appendix 01 (PDF)

## Data Availability

All study data are included in the article and/or *SI Appendix*.
