# Supplementary material for: Biomass-burning organic aerosols as a pool of atmospheric reactive triplets to drive multiphase sulfate formation
Source: Proc Natl Acad Sci U S A. 2024 Dec 13;121(51):e2416803121. doi: 10.1073/pnas.2416803121 (PMC11665901; doi:10.1073/pnas.2416803121)
Supplement: Supplementary file 1 — Appendix 01 (PDF) [file pnas.2416803121.sapp.pdf]

PNAS

Supporting Information for

Biomass Burning Organic Aerosols as a Pool of Atmospheric Reactive Triplets to Drive Multiphase Sulfate Formation

Zhancong Liang, Liyuan Zhou, Yuqing Chang, Yiming Qin, Chak K. Chan*

*Corresponding author: Chak K. Chan

Email: chak.chan@kaust.edu.sa

This PDF file includes:

Text. S1 to S10

Figs. S1 to S26

Tables S1 to S5

References

Supplementary texts

Text S1. Estimation of the pH of BBOA particles.

Laser spectroscopic methods, such as Raman and fluorescence lifetime imaging, cannot be applied to BBOA particles due to their light-absorbing nature (1-4). Another option is colorimetric analysis using pH-indicator papers (5, 6), though this method can be interfered with by the inherent color of BBOA. To address this, we developed a pH calibration curve following Craig, *et al.* (5) and examined the potential effect of colored particulate components. In brief, we prepared malonate buffer solutions with varying ratios of malonic acid and malonates, atomized them into droplets, pre-equilibrated them at 80% RH, neutralized them, and sized them to 100 nm. These droplets were then collected on pH indicator paper in an aerosol impactor for around 30 minutes. The RGB values from the standard color bar were extracted, and a calibration curve between ‘G minus B’ values and the corresponding pH was established.

Next, we imaged the particle-loaded pH indicator papers, extracted the RGB values, and used the calibration curve to calculate pH. To assess the effect of BBOA's inherent color on colorimetric pH determination, we spiked SinAld, a non-dissociable compound, into the buffer solution until its mass absorption coefficient matched that of the BBOA solution used for atomization. The choice of SinAld was driven by its greatest contribution to the light-absorption of BBOA among different identified chromophores based on HPLC-PDA (Figure 4A). IC and HPLC analyses indicated that the difference in [SinAld]/[malonates] was less than 7% between the solution used for atomization and the extract of collected particles. This suggests minor evaporation of SinAld, as malonates can be considered less volatile. Lastly, we found that the presence of SinAld led to an underestimation of pH by 0.3 ± 0.2 units in the pH range of 2 to 7 (Figure S4).

After correction, the pH of BBOA particles at 80% RH was measured as 4.4, comparable to the value estimated using charge conservation. Thus, a ‘pH-shift’ is not a significant contributor to the observed high sulfate formation rate in BBOA particles, as a pH of 7 would be required for such a scenario.

Text S2. The formation of OH• in BBOA.

Benzoic acid (BA) was spiked into the BBOA solution as a chemical probe to determine OH• concentration based on competition kinetic (7). The mixture of BA and BBOA was adjusted to pH 4.2 using H_2SO_4 and NaOH, and subsequently illuminated by UVA. Solutions after different illumination times were sampled. The HPLC-PDA was used to monitor the formation of p-hydroxybenzoic acid (p-HBA, formed via OH• oxidation of BA).

The concentration of p-HBA as a function of illumination time was fitted by:

$$[p-HBA]_t = [p-HBA]_0 + a(1-e^{-bt}) \quad \text{(Eq.1)}$$

The initial formation rate of p-HBA (R) can be calculated with:

$$R = a \times b \quad \text{(Eq.2)}$$

Then, the 1/R was fitted versus 1/[BA] using a linear regression. The formation rate ($F_{OH\cdot}$) and pseudo-first-order sink rate of OH• ($k'_{OH\cdot}$) can be calculated as:

$$F_{OH\cdot} = \frac{1}{y\ intercept \times Y_{p-HBA}} \quad \text{(Eq.3)}$$

$$k'_{OH\cdot} = k_{OH\cdot,BA} \times (\frac{slope}{y\ intercept}) \quad \text{(Eq.4)}$$

$Y_{p\text{-}HBA}$ is the yield of p-HBA formation from OH oxidation (0.18). $k_{OH\cdot,\ BA}$ is the second-order reaction rate constant between OH• and BA at pH of 4.2 (5.1×10^9 M^{-1} s^{-1}) (8).

At the mass ratio of BBOA to the water of 1, the [OH•] was estimated as 10^{-18} M (Eq.5), which is six orders of magnitude lower than the [$^3BBOA^*$].

$$[OH\ \cdot] = \frac{1}{k_{OH\cdot,BA} \times slope \times Y_{p-HBA}} \quad \text{(Eq.5)}$$

Text S3. The electron transfer of DMB at the air-water interface and bulk.

Molecular dynamic simulation coupled with quantum chemical calculation was used to illustrate the difference between electron-transfer reaction for DMB at the interface and in the bulk, from a perspective of molecular energy. In brief, the geometry optimizations were conducted using Gaussian 16, based on the B3LYP functional complemented by the 6-31G* basis set and enhanced with Grimme D3 dispersion corrections applying Becke-Johnson damping factors. Harmonic vibrational frequency calculations were performed to confirm the stationarity and nature of the points as true minima or transition states, while also providing necessary thermodynamic corrections to the SCF energies. An explicit solvent model for the bulk phase and the water-air interface was created. Specifically, the solute system was encapsulated within a solvent sphere using Packmol, followed by a preliminary optimization. This setup was further refined by extracting the solute with a solvent layer using Visual Molecular Dynamics (VMD) software and optimizing it with MOPAC 2016. The system stability was finalized through density functional theory (DFT) methods in Gaussian 16. The last frame of the molecular dynamic simulation structure was sampled and optimized via DFT to yield the energy of a stable structure. Moreover, electron localization field (ELF) diagrams were generated using Multiwfn software to visualize the electron distributions (9), as shown in Figure S7.

Text S4. The calculation of the energy and one-electron reduction potential for different $^3C^*$s.

The triplet energy (E_T) and one-electron reduction potential (E^o, SHE) of photosensitizers were calculated by Gaussian 16. The time-dependent density functional theory (TD-DFT) calculations were used to determine E_T, based on M06-2X functional with the cc-pVTZ basis set, which has been considered one of the best functions for excitation energy calculation (10). All E_T was taken the $T_1 \rightarrow S_0$ transitions.

Moreover, the E^o for the ground state photosensitizers was carried out with the M06-2X-D3/def2-SVP level of theory, according to:

$$E^o{}_{Ground} = \frac{(G_{Gound} - G_{One-electron-reduced})}{nF} - 4.281 \quad \text{(Eq.6)}$$

n is the number of electrons transferred, which is 1. F is the Faraday constant 96.485 kJ V^{-1}. 4.281 V is the absolute potential of the standard hydrogen electrode (SHE). Gibbs free energy (G) calculations on the equilibriums of the ground state and one-electron reduced state were carried out by performing frequency calculations to derive the enthalpy and entropy values. The solvation model based on density (SMD) implicit solvation model of water was employed to account for the solvent effect (11). Then, the E^o for the triplet excited states was calculated by:

$$E^o = E^o{}_{Ground} + \frac{E_T}{F} \quad \text{(Eq.7)}$$

The E^o and E_T are summarized in Table S2.

Text S5. Estimation of the sulfate formation due to $^3BBOA^*$ in wildfire-prone regions.

We used a simplified scheme to estimate the impact of BBOA photochemistry on air quality in wildfire-prone regions, using sulfate formation as an indicator. We used data from the NASA fire map (https://firms.modaps.eosdis.nasa.gov/) to determine areas most affected by wildfires and compared the sulfate formation rates from $^3BBOA^*$ pathways with those resulting from H_2O_2 oxidation, which is a dominant global mechanism (12). We calculated sulfate formation rates using the following equation:

$$\frac{d[SO_4^{2-}]}{dt}(\mu g\ m^{-3}\ h^{-1}) = 3600\ (s\ h^{-1}) \times 96\ (g\ mol^{-1}) \times \frac{C}{\rho}\ (\frac{mg\ m^{-3}}{kg\ L^{-1}}) \times R\ (M\ s^{-1}) \qquad \text{(Eq.8)}$$

where 3600 s h^{-1} is a time conversion factor. 96 g mol^{-1} is the molar mass of SO_4^{2-}. L is the BBOA particle concentration for the $^3BBOA^*$ pathway or aerosol water content for the H_2O_2 pathway, mg m^{-3}. ρ is the density of BBOA (1.3 g cm^{-3}) (13) or water (1 g cm^{-3}). R is the overall sulfate formation rate in BBOA or aerosol liquid water, measured in M s^{-1}.

The sulfate formation rate by H_2O_2 in aerosol liquid water can be expressed as:

$$R_{H_2O_2} = \frac{k_{H_2O_2,S(IV)}[H^+][HSO_3^-][H_2O_2(aq)]}{1+K[H^+]} \qquad \text{(Eq.9)}$$

$k_{H_2O_2,S(IV)}$ and K are 7.45×10^7 M^{-1} s^{-1} and 13 M^{-1}, respectively (14). We used the $R_{^3BBOA^*}$ in 100 nm particles in this study (15). For our scenarios of varying wildfire severity, we used concentrations of 50 and 20 μg m^{-3} $PM_{2.5}$ to model severe and moderate wildfire conditions, respectively. The concentration of background aerosol was assumed as 10 μg m^{-3}. The severe wildfire scenario is intended to reflect potential future hazards from intensified wildfires (16).

The production rate of $^3BBOA^*$ depends on the actinic flux. There, we scaled the [$^3BBOA^*$] at different locations using their corresponding actinic flux calculated by the TUV model (https://www.acom.ucar.edu/Models/TUV/Interactive_TUV/). In the TUV model, the overhead ozone column was fixed at 300 du. Wildfire was reported to deplete ozone, thus our calculated actinic flux may have been underestimated (17, 18). The surface albedo at the UV range was fixed at 5% (19). Both the ground elevation and measure altitude are 0 km asl. The optical depth, base, and top for clouds are 0, 4, and 5, respectively. The direct beam, diffuse down, and diffuse up input

for sunlight are all set to 1. The input parameters for aerosol are varied by scenarios. Typical levels for aerosol optical depth (AOD) at 550 nm can span a wide range during a wildfire, complicated by the distribution of BBOA and other parameters such as temperature (20). However, the AOD exhibited an overall positive correlation with particle concentration, and we simplified the AOD to 0.5 and 0.2 at 50 and 20 μg m^{-3} of $PM_{2.5}$ (21-23).

The single-scattering albedos (SSA) and alpha (Angstrom exponent, AE) were estimated as:

$$SSA = \alpha_{BBOA} \times SSA_{BBOA} + \alpha_{BG} \times SSA_{BG} \quad \text{(Eq.10)}$$

$$AE = \alpha_{BBOA} \times AE_{BBOA} + \alpha_{BG} \times AE_{BG} \quad \text{(Eq.11)}$$

α_{BBOA} and α_{BG} denote the mass concentration fraction of BBOA and background aerosols. SSA for BBOA and background PM are 0.85 and 0.99, respectively (15). AE for BBOA and background PM are 3.3 and 1, respectively (24).

All parameters for sulfate formation rate estimation were summarized in Table S3. Since BBOA themselves are the major light absorbers that affect the actinic flux, therefore the scaling of [$^3BBOA^*$] by the actual actinic flux might underestimate the BBOA photochemistry. For the sulfate formation rates, the [S(IV)] and [H_2O_2 (aq)] were calculated by the atmospheric concentrations obtained from the GEOS-Chem simulation reported by Gao, *et al.* (12) and their Henry's law constants. The [H^+] was derived from the thermodynamic simulation using ISOPPOPIA II, referenced from the same study. Wildfires exhibited strong seasonality with summer as the peak period due to the high temperature and drought (25). Therefore, our analysis specifically utilized simulation results from July 2019 for all regions except for Australia, which has wildfire peaked in Oct 2019.

Text S6. The HPLC-PDA-HRMS analysis of BBOA.

The molecular identity, light-absorbing properties, and relative abundance of brown carbon chromophores were determined using a high-performance liquid chromatograph-photo diode array-high resolution mass spectrometer (HPLC-PDA-HRMS, Orbitrap Exploris 120, Thermo Fisher). A reverse-phase column (ACE Excel 5, C18-AR, 4.6×250mm, 5 µm particles, 100 Å, VWR) was used for LC analysis. The injection volume was 20 µL. The mobile phase comprised 1% formic acid in LC-MS grade water (A) and acetonitrile (B). Gradient elution was performed with the mixture of A-B at a flow rate of 1 mL min^{-1}: 0–3 min holds at 5 % B, 3–12 min linear gradient to 60 % B, 12– 20 min linear gradient to 5 % B, 20–25 min holds at 5% B, 25-26 linear gradient to 5 % B, 26–30 min holds at 5 % A. The PDA was operated at full wavelength analysis at 200-800 nm, with an interval of 2 nm.

The orbitrap analysis was performed at heated electrospray ionization (H-ESI), with 3.5 kV and 2.5 kV spray voltage for positive ion and negative ion, respectively. The flow parameters are 60 units of sheath gas flow, 15 units of auxiliary gas flow, and 2 units of sweep gas flow. The ion transfer tube temperature and vaporizer temperature are both 350 ^{o}C. During the analysis, the temperature of the sample rack was kept at 4 ^{o}C to minimize the potential sample degradation. To avoid the residual analytes interfaces in the MS analysis of the next samples, the elute from LC was directed to the waste in 0-2 min and 25-30 min. The HPLC-PDA-HRMS data was analyzed using Xcalibur software. The chemical formulas were determined from the exact m/z values using the formula calculator incorporated in Xcalibur, to infer the chemical structure. Then, standard chemicals were used for identification.

Text S7. The GC×GC ToF MS analysis of the hexane-extract of biomass burning particles.

GC×GC MS analysis was carried out using an Agilent 8890A GC × GC coupled with an Agilent 7250A-HES TOF mass spectrometer. DB-5MS (60m*0.25mm*0.25μm), DB-17MS (0.85m*0.25mm*0.15μm), and C7-C40 were used as the first- and second-dimension GC columns and the modulation column, respectively. The GC chamber was first stabilized at 50 ^{o}C for 2 min, then increased to 300 ^{o}C with a speed of 3 ^{o}C min^{-1}, followed by staying at 300 ^{o}C for 5 min. Helium (99.999%) was used as the carrier gas at a speed of 1.3 mL min$_{-1}$. The temperature of the injection port was 300 ^{o}C and the injection volume was 1 μL.

The entrance and exit temperatures of the modulator (SSM1810, Xuejing Scientific) were +0 ^{o}C and +30 ^{o}C, relative to that of the GC chamber. The temperature of the cooling zone was -51 ^{o}C and the modulation period was 4 s. The temperature of the ionizer is 210 ^{o}C and the ionization temperature is -70 eV. The detected mass spectral range was m/z 40-650, and the spectra acquisition rate of 50 spectra per second. The data were analyzed by Canvas, and the compound databases such as NIST17 were used to identify compounds.

Text S8. The morphology and spatial distribution of the chemical components within the BB particles

After a two-stage dilution of dilution, the BB particles were directly sampled onto the hydrophobic and oleophobic surface, using an aerosol impactor (TSI) at a cut size of around 0.3 μm, for ∼3 min. Collection times were long enough so that submicron BBOA particles deposited and agglomerated on the slides to form super-micron BBOA particles. The silicon wafer with BB particles loaded was then moved to a customized quartz flow cell with a CaF_2 window, which allows the penetration of the Infrared laser beam. The flow cell was continuously purged with a dry-wet mixed flow of N_2 to minimize the photobleaching of chromophores in BB particles and maintain a relative humidity (RH) of 80%. A Vaisala RH sensor was used to monitor the RH of the flow at the exhaust of the flow cell.

The O-PTIR and confocal Raman imaging was performed using a mIRage infrared + Raman microscope (Photothermal Spectroscopy Corp.). The mIRage contains a custom microscope frame with a visible objective and a Cassegrain reflective objective for the simultaneous use of IR and visible lasers. A continuous-wave laser source (532 nm) was used as the probe, while two pulsed and tunable infrared lasers (a quantum cascade laser (QCL) to cover 880–1950 cm^{-1}, and an optical parametric oscillator (OPO) to cover the range 2700–3600 cm^{-1}) to generate photothermal enhancement. Before imaging, we optimized the laser focus for each QCL chip in 1726, 1575, 1270, and 1026 cm^{-1} using the IR signal from a polyethylene terephthalate standard within the instrument. IR spectra were collected at a scan rate of 100 cm^{-1} s^{-1} for 3 s acquisitions and averaged after ten accumulations. Raman scattering was detected after photons passed back through the Cassegrain objective and a pinhole, into a Horiba iHR320 module (focal length = 320 mm). PTIR Studio software (version 4.0, Photothermal Spectroscopy Corp.) was used to process spectra and IR images.

Text S9. The photon flux in the photochemical reactors.

A chemical actinometer 2-nitrobenzaldehyde (2NB) was used to determine the photon flux in the AFT and aqueous reactor. For the AFT, a quartz cuvette containing 50 μM 2NB water solution was placed at the axial line of the flow reactor. An aliquot was sampled from the illuminated solution every 1 minute to measure the concentration of 2NB using HPLC-PDA. The channel with UV absorption at 254 nm was used to quantify 2NB after calibration. The photolysis rate constant (i.e., j(2NB)) can be retrieved from the 2NB decay kinetic, according to:

$$j(2NB) = -\frac{\ln\left(\frac{[2NB]_t}{[2NB]_0}\right)}{t} \quad \text{(Eq.12)}$$

where $[2NB]_t$ and $[2NB]_0$ are the 2NB concentrations at time t and 0, respectively. Then, the photon flux can be obtained according to:

$$j(2NB) = 2.303 \times \frac{10^3}{N_A} \times \sum(I_\lambda \times \Delta_\lambda \times \varepsilon_{2NB,\lambda} \times \Phi_\lambda) \quad \text{(Eq.13)}$$

where N_A is Avogadro’s number, I_λ is the actinic flux, Δλ is the wavelength interval between actinic flux data points, $\varepsilon_{2NB,\lambda}$, and Φ_{2NB} are the base-10 molar absorptivity and quantum yield for 2NB, respectively. $\varepsilon_{2NB,\lambda}$ at each wavelength under 298 K, and a wavelength-independent Φ_{2NB} value of 0.41 were adapted from the literature (26). The transmission (%) of our quartz glass tube used for photon flux determination is stable from 300 to 420 nm with a high value of around 90%. Therefore, we corrected the photon flux by dividing 0.9. All reported kinetic and concentration data in this work have been normalized to sunlight conditions at noon time on July 1st in British Columbia, Canada, calculated by the TUV model. The actinic flux in the aqueous reactor was determined by monitoring the decay of 2NB spiked into the BBOA solution, following the same protocol. We did not expect the internal screening effect (or shielding effect) in our BBOA particles in the AFT due to the small size (27). However, 2-NB in the aqueous reactor was subjected to the internal light screening effect by the BBOA chromophores. Therefore, we corrected the internal light screening effect according to Smith, Kinney and Anastasio (28) for results from aqueous reactors.

Taking a [^{3}BBOA*] of 10^{-12} M and a maximum second-order reaction rate constant of 10^{10} M^{-1} s^{-1}, the pseudo-first-order reaction rate constant between ^{3}BBOA* and 2NB can reach 10^{-2} s^{-1}, at the comparable level of the photolysis rate constant of 2NB. Therefore, the photon flux to the aqueous reactor can be overestimated, leading to an underestimation of the [^{3}BBOA*] and sulfate formation rate when normalized to ambient conditions.

Text S10. The mass loading of collected particles.

To quantify the particle mass collected on Teflon filters, we used an ultrasensitive microbalance (Zhanhai) housed within a glove box where both temperature and relative humidity (RH) were controlled. The temperature was maintained at 293 K, and RH was adjusted to match the conditions under which the samples were collected., to minimize the evaporation loss of organics. For calibration of the microbalance, we utilized a protocol involving the generation of ammonium sulfate and ammonium nitrate particles through atomization. These particles were conditioned at various RH levels and passed through the AFT system before being collected on the Teflon filter. After collection, the loaded filter was immediately weighed on the microbalance under the same RH conditions to ensure accuracy. At a parallel stream to the filter collection, a Scanning Electrical Mobility Spectrometer (SEMS, BMI) was used to measure the size distribution of the particles to retrieve the loading on the filter and compare it with the weighing results, shown in Figure S25.

Supplementary Figures

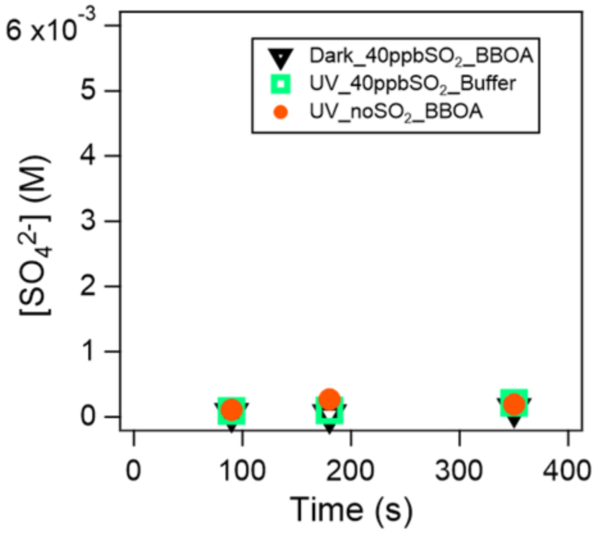

Fig. S1. Sulfate formation under dark and via photochemistry of S(IV) and organic sulfur compounds. The sulfate concentration as a function of time for SO_2 aging of BBOA particles under dark conditions (dark triangle), SO_2 aging of organic buffer particles under light (green square), and aging of BBOA in the absence of SO_2 under light (organic sphere). The SO_2 concentrations are 40 ppb if applicable. The RHs are 80%.

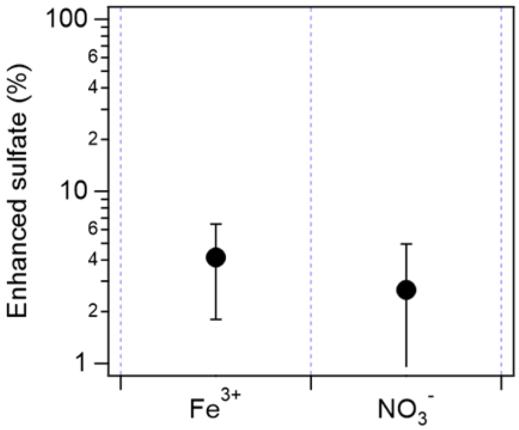

Fig. S2. The role of iron and nitrate photochemistry. The percentage of enhanced sulfate formation after doubling the concentrations of Fe^{3+} and NO_3^- in the BBOA solution used for generating particles. $FeCl_3$ and KNO_3 were used as the source of Fe^{3+} and NO_3^-.

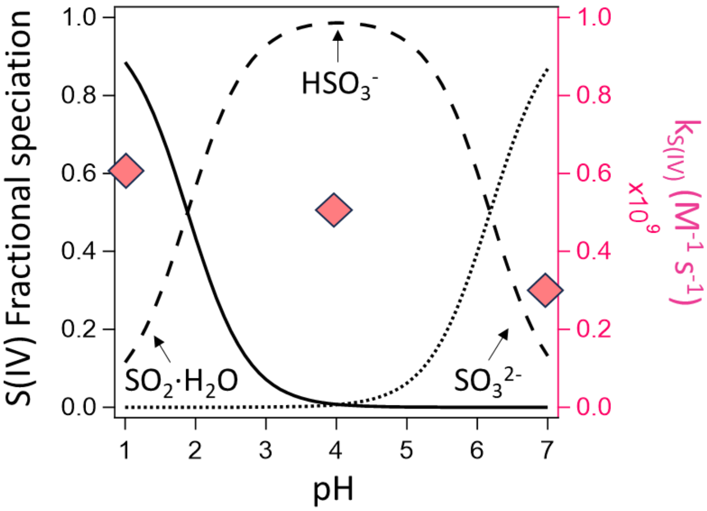

Fig. S3. The speciation of S(IV) and quenching rate constant of [3]BBOA* by S(IV). The pHs were 1, 4, and 7. The quenching rate constants were derived from TA measurements using BBOA solutions.

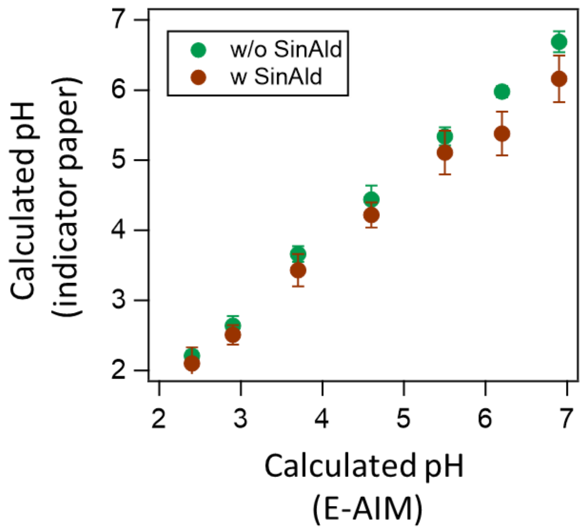

Fig. S4. The pH measurements for particles with and without brown carbon. The relationship between calculated pH by indicator paper methods and by E-AIM model.

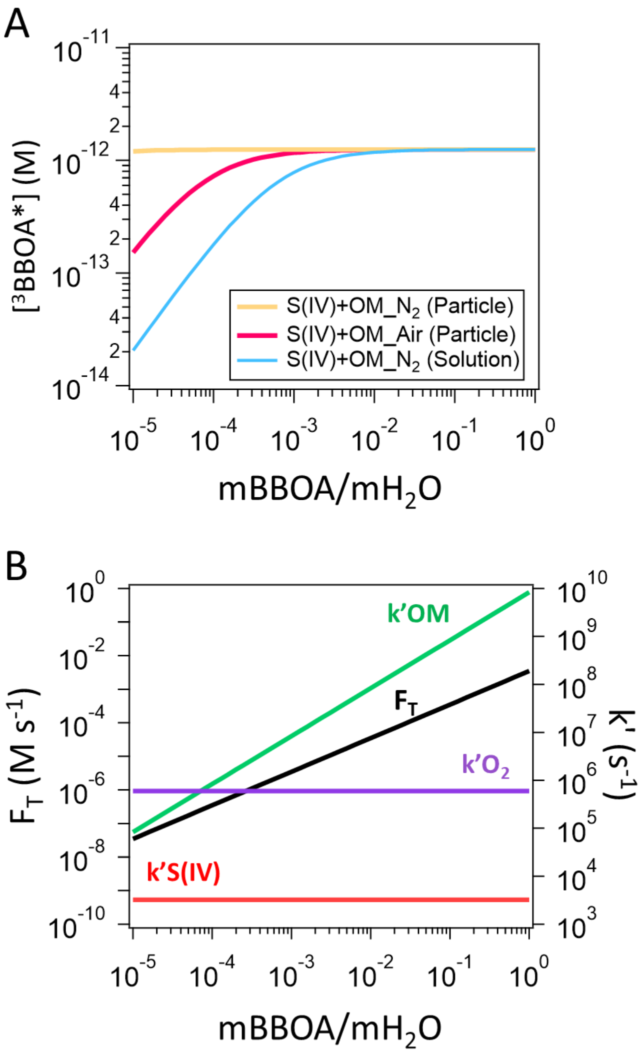

Fig. S5. The modeling of ^{3}BBOA* concentrations. (A) The modeled concentration of 3BBOA* in BBOA particles by considering different sinks. The initial S(IV) concentration in solution is 10 mM; **(B)** The fractional speciation of S(IV) (left panel) and second-order quenching rate constant between ^{3}BBOA* and S(IV) (right panel) at different pHs. The quenching rate constants were derived from TA measurements using BBOA solutions.

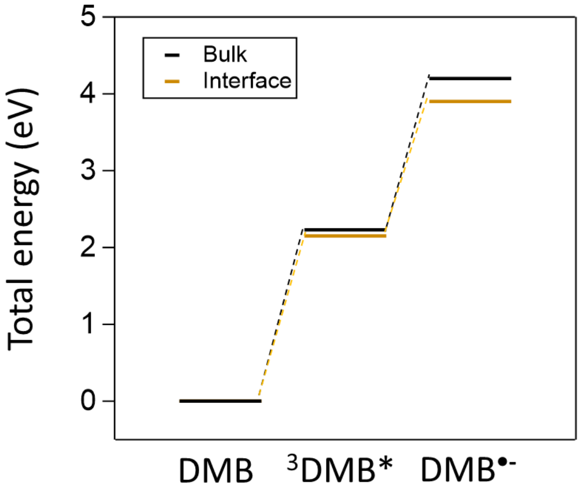

Fig. S6. The total energy for DMB,^{3}DMB*and DMB$^{\bullet-}$at the interface and in bulk. The calculation method was detailed in Text S3.

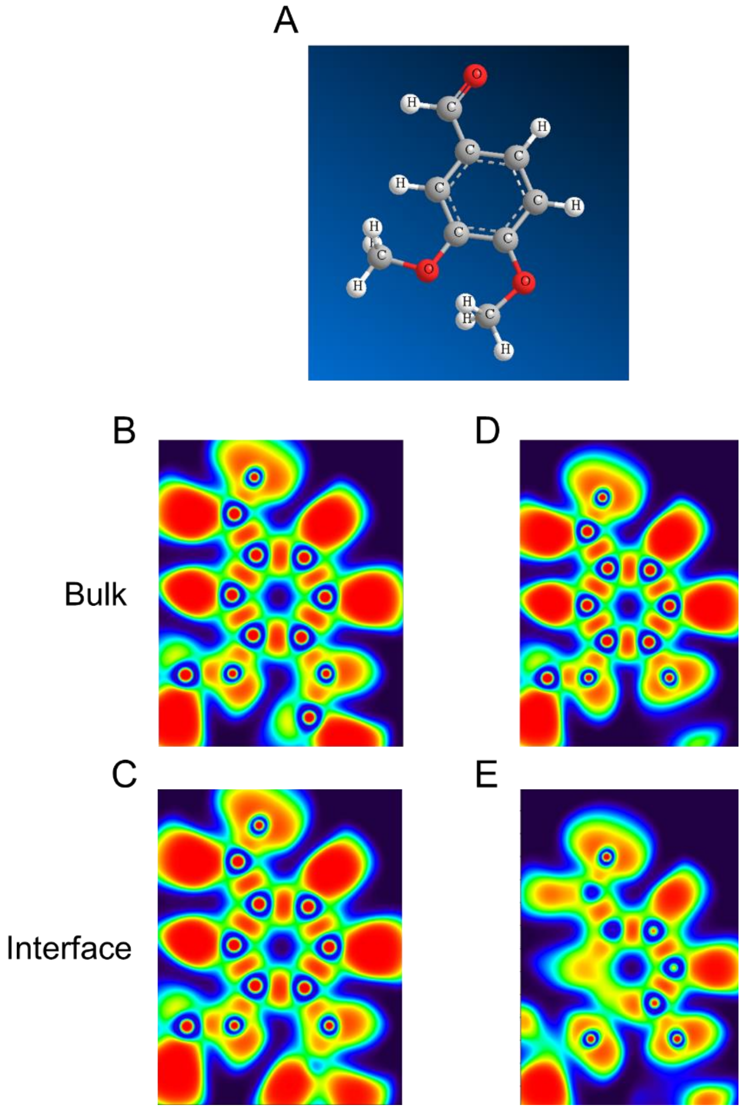

Fig. S7. The ELF of ^{3}DMB* and (D) DMB$^{\bullet -}$. (A) The structure and elemental composition of DMB; The ELF map of **(B)** ^{3}DMB* and **(D)** DMB$^{\bullet -}$ in the bulk phase, and the ELF map of **(C)** ^{3}DMB* and **(E)** DMB$^{\bullet -}$ at the air-water interface.

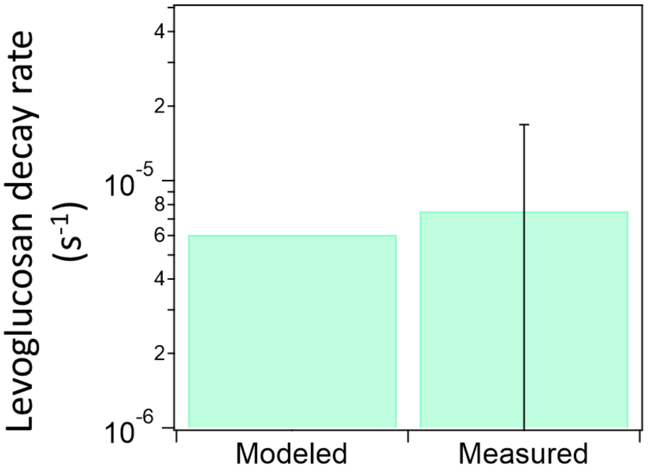

Fig. S8. The model and measured decay rate of levoglucosan in BBOA particles. The initial particle sizes of 100 nm. The RH is 80%. Due to the slower reactions in the BBOA bulk, we measured the levoglucosan decay under 20× light intensity by increasing the number of the light tubes and then normalized to 1× condition.

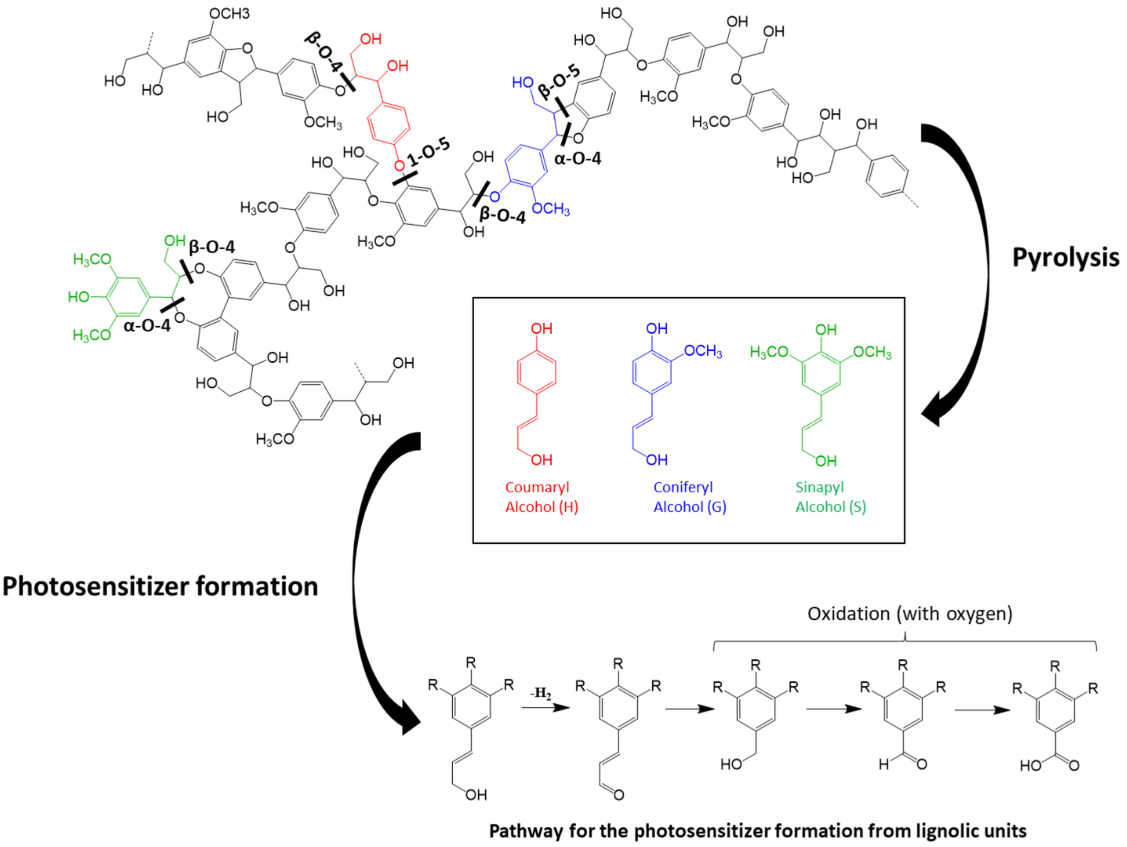

Fig. S9. The formation mechanism of photosensitizers. The formation of conmaryl, coniferyl, and sinapyl units via pyrolysis of lignin and further conversions to photosensitizers through oxidative pyrolysis.

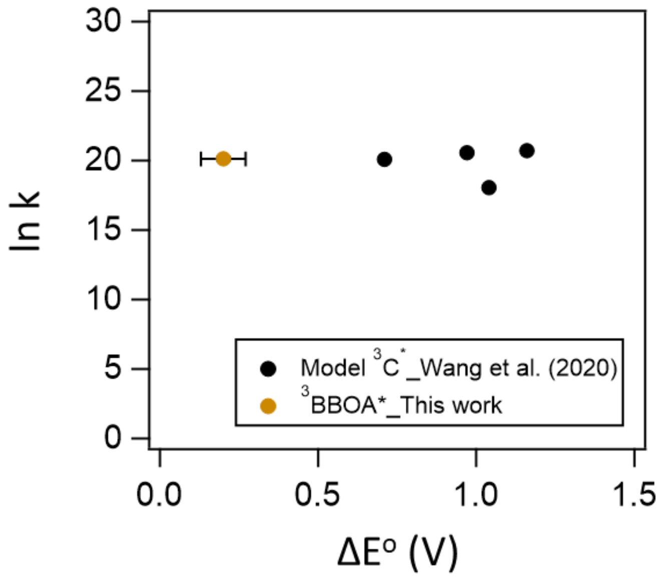

Fig. S10. The dependence of ln k of $^3C^*$ quenching by S(IV) on ΔEº. The logarithm of the second-order quenching rate constant of $^3C^*$ by S(IV) and the difference between the one-electron reduction potential of $^3C^*$ and the oxidation potential of S(IV). The quenching rate constants at pH 1 and 4 were averaged to compare with Wang, *et al.* (29) at pH 1.8.

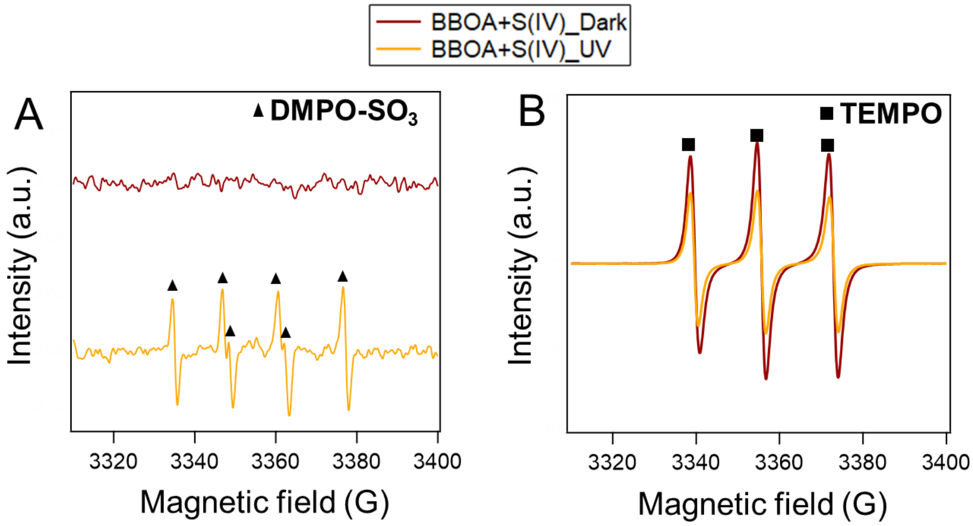

Fig. S11. The electron-transfer from S(IV) to ^{3}BBOA*. The EPR spectra of the BBOA and $NaHSO_3$ mixture with (A) DMPO and (B) TEMPO under dark and UV after 30 min. The triangle labels the fingerprint of DMPO-SO_3.

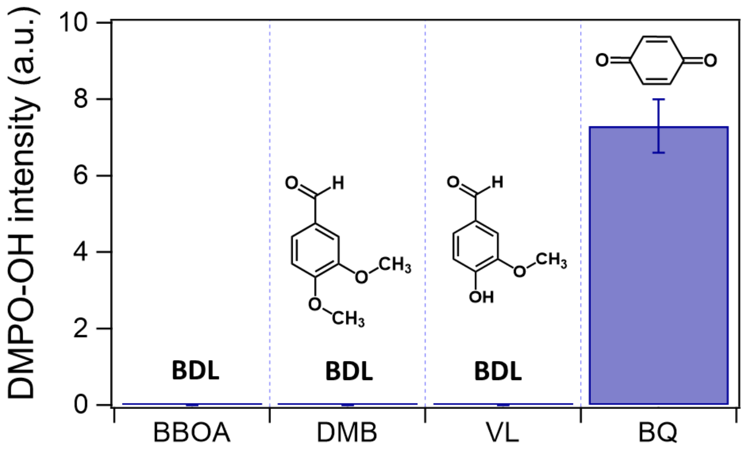

Fig. S12. The intensity of DMPO-OH absorption peaks in the EPR spectra. The illumination times are 30 min. BDL denotes below the detection limit of around 10^{-7} M. The concentrations of photosensitizers and BBOA are all 10 mg L^{-1}, and the concentration of DMPO is 0.1 M. The solutions have been deoxygenated by purging N_2 for 30 min and sealed before in-situ EPR measurements.

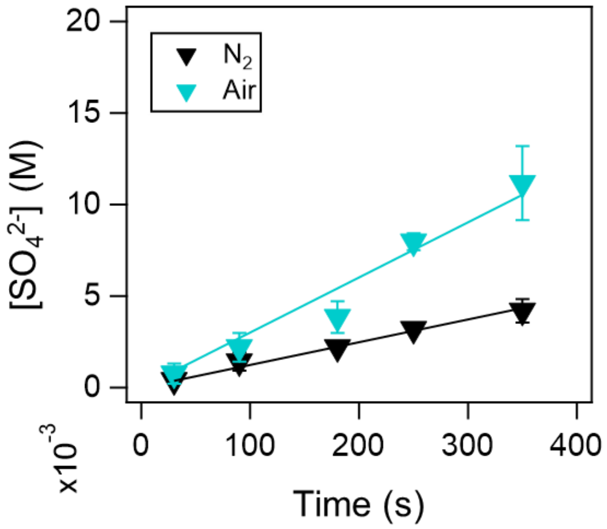

Fig. S13. Photosensitized sulfate formation in BBOA in air. The sulfate concentration in BBOA as a function of illumination time under air and N_2 conditions. The SOA concentration is 40 ppb and RH is 80%. The initial size of particles is 100 nm. The light intensity is 100%.

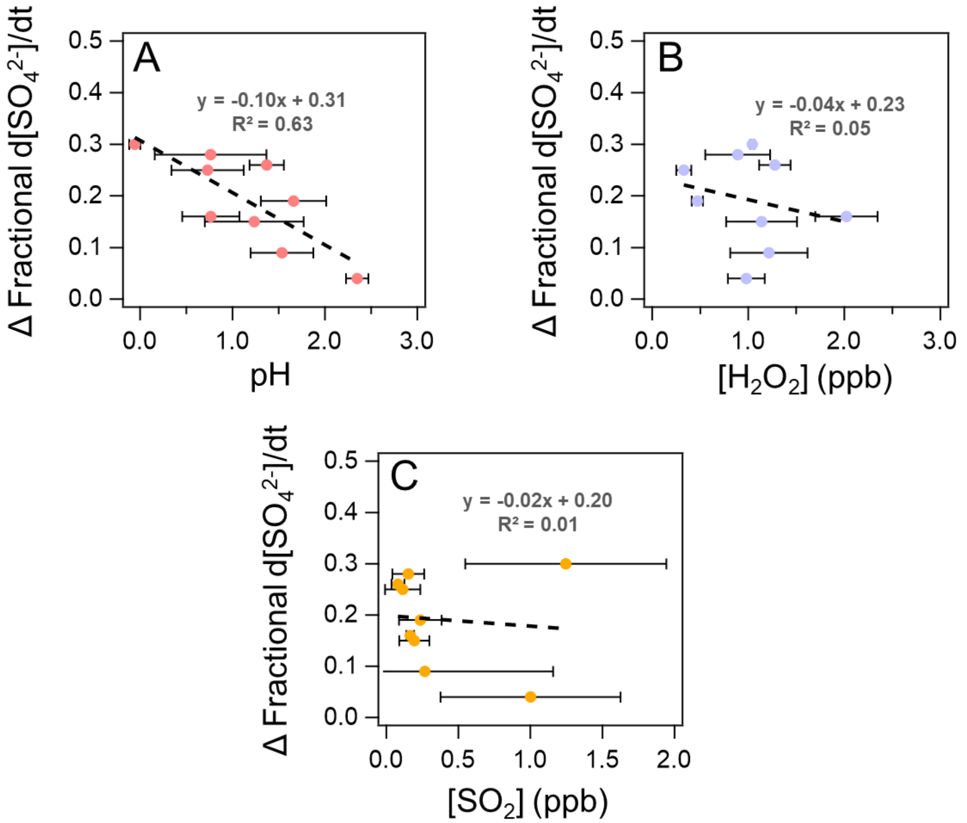

Fig. S14. The correlation between sulfate formation by ³BBOA* and environmental conditions. The increase in fractional sulfate formation rate upon moderate fire transit to severe fire as a function of **(A)** pH, **(B)** [H_2O_2], and **(C)** [SO_2], across various global regions. The dashed lines show the linear regression curves.

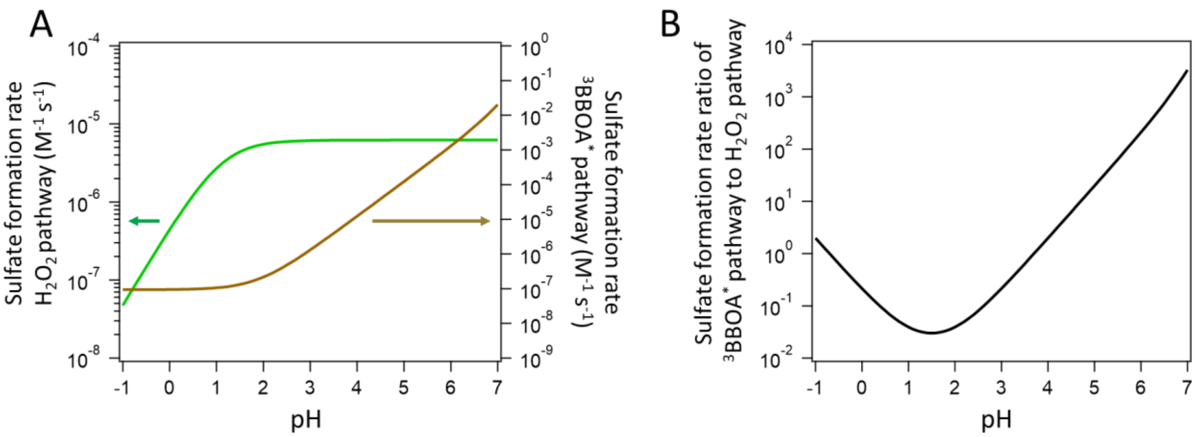

Fig. S15. The pH-dependence of sulfate formation. (A) The calculated sulfate formation rate via the H_2O_2 pathway (left axis) and ^{3}BBOA* pathway (right axis) as a function of pH. The [SO_2] is 40 ppb, a typical level for urban haze in China (*16*); **(B)** The sulfate formation rate ratio of ^{3}BBOA* pathway to H_2O_2 pathway as a function of pH.

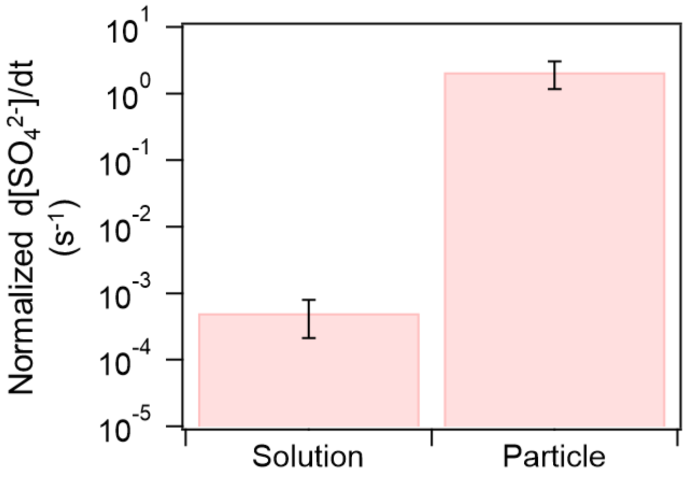

Fig. S16. Enhanced sulfate formation by ^{3}2-IC* in particles over solution. The normalized sulfate formation rate in mixed particles and solution of 2-IC and the organic buffer. Both aerosol particles and bulk solutions contain 1 mM photosensitizers and around 3 M sodium bimalonate/sodium malonate=1:1 buffer.

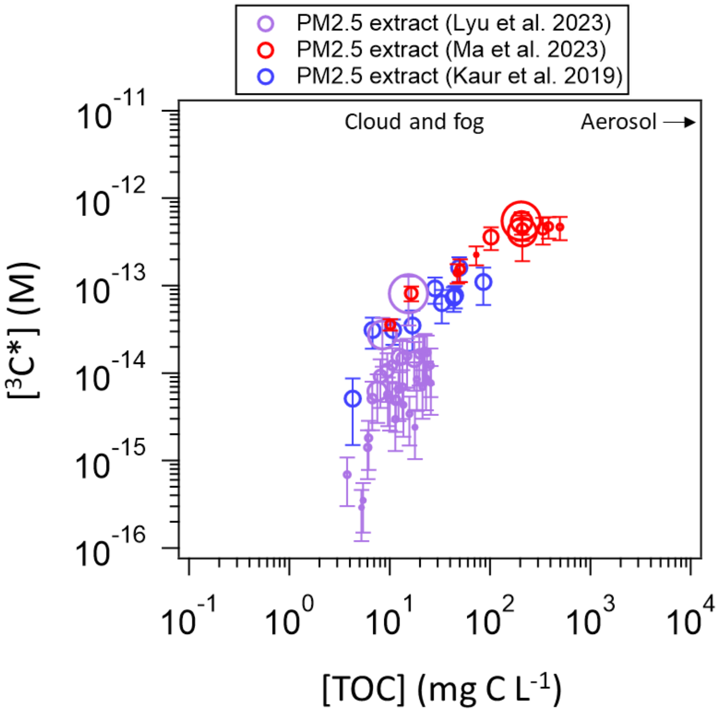

Fig. S17. The reported concentration of $^3C^*$ in the illuminated extract of ambient particles. The data are sourced from (7, 30, 31). Given that the photon flux at typical Beijing haze conditions is attenuated by around 70% compared to clear days, 10^{-13} M of $^3C^*$ was used for discussions in the main text.

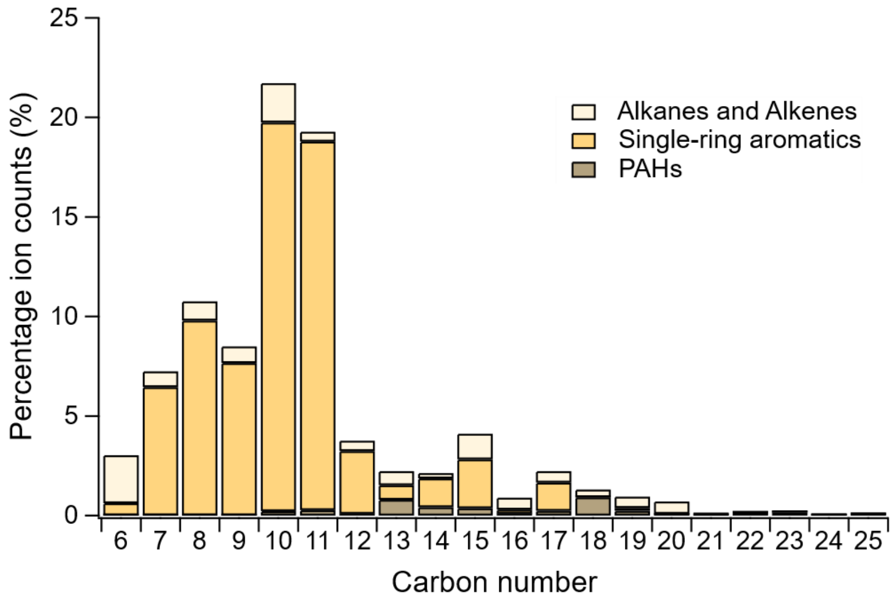

Fig. S18. The composition of hexane extract of BBOA by GC×GC ToF MS analysis. The percentage ion counts from GC×GC ToF MS analysis as a function of carbon number were shown. Formulars of C_xH_{2x+2} - $_{2x-4}$ were assigned as alkanes and alkenes, C_xH_{2x-6} - $_{2x-10}$ were assigned as single ring aromatics, C_xH_{2x-12} – $_{2x-20}$ were assigned as poly aromatic hydrocarbons (PAHs), according to He, *et al.* (32).

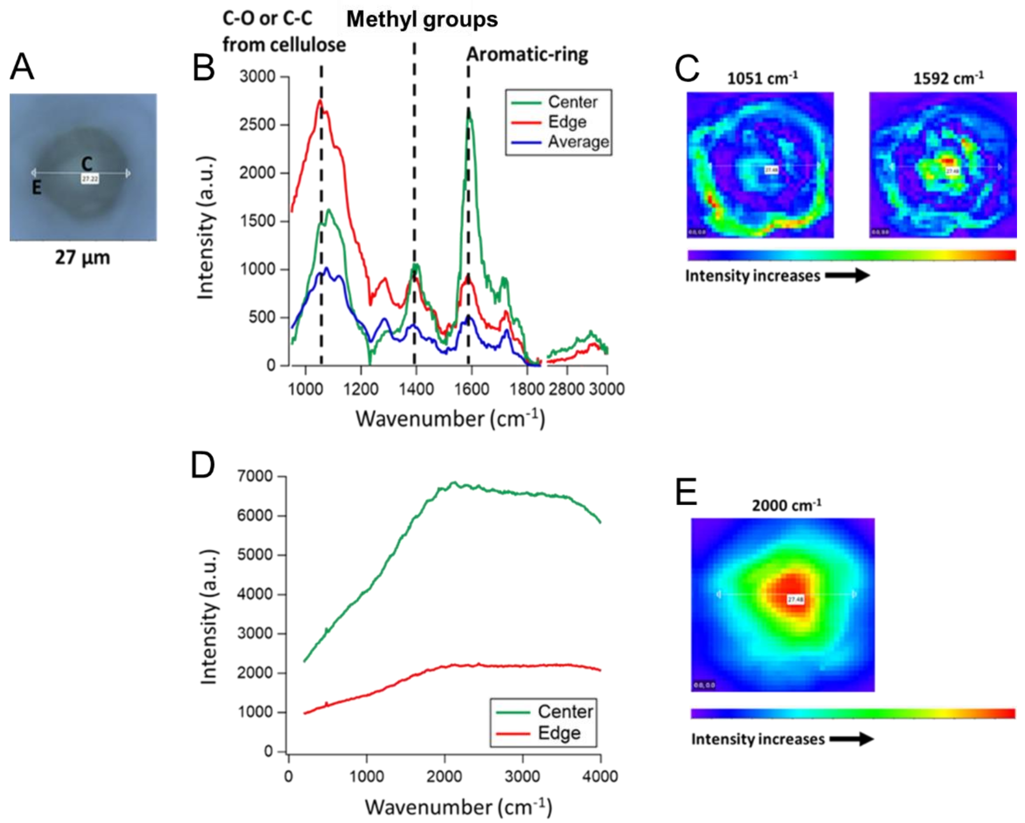

Fig. S19. The phase separation in BB particles. (A) The optical image of the BB particles; **(B)** The average O-PTIR spectra of the BB particles and the spectra at the center (C in Figure A) and the edge (E in Figure A); **(C)** The spatial distribution of the IR characteristic peak at 1051 cm^{-1} and 1593 cm^{-1}; **(D)** The Raman spectra at the center and edge locations, and **(E)** the distribution of Raman characteristic peak at 2000 cm^{-1}.

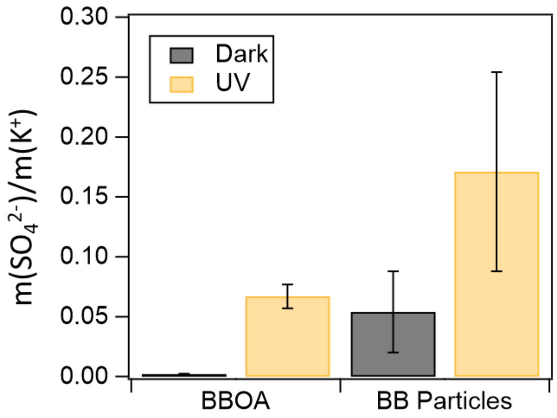

Fig. S20. Photosensitized sulfate formation in BBOA particles in N_2. The mass ratio of SO_4^{2-} to K^+ in BBOA and BB particles under dark and light conditions. BB particles denote those directly emission from the tube furnace without being extracted by solvent, while BBOA particles are atomized from the water-extract of the collected BB particles. The SO_2 concentrations are around 40 ppb, and RHs are around 80%.

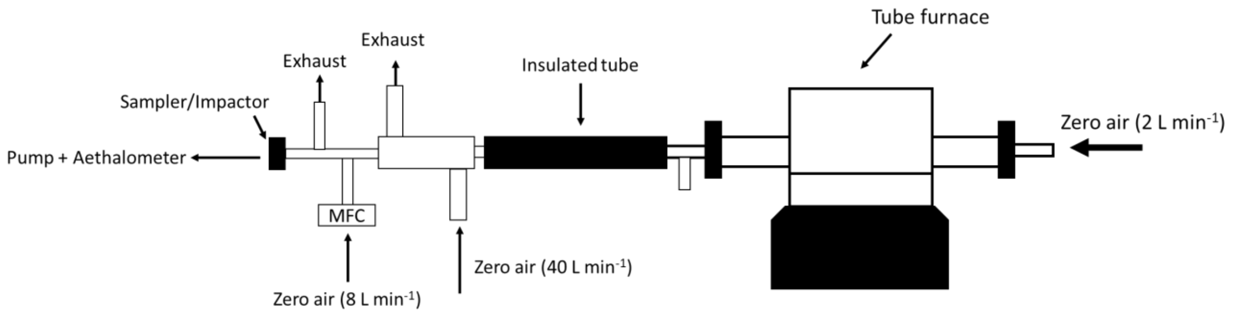

Fig. S21. The schematic for biomass burning particle generation system. The dilution factor is around 80, which falls between that for small agricultural prescribed burn (1.8×10^5) and large wildfires (1.3) (33).

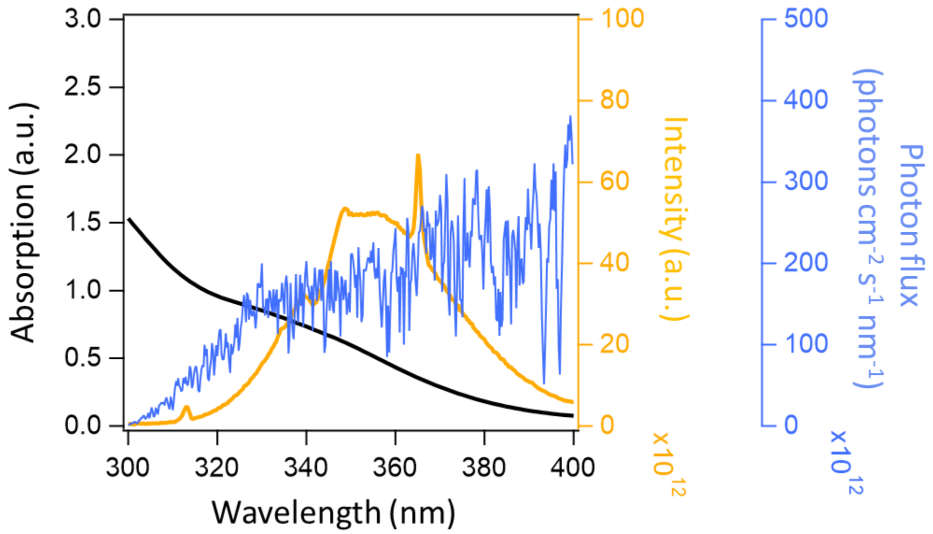

Fig. S22. The absorption spectra of BBOA and emission spectra of light tubes and sunlight. The light absorption spectra of the BBOA from 300-400 nm (left axis), the emission spectra of the UVA light used for photochemical experiments (right yellow axis), and the calculated photon flux in British Columbia, Canada at noon time on July 1st, 2019 (Blue axis).

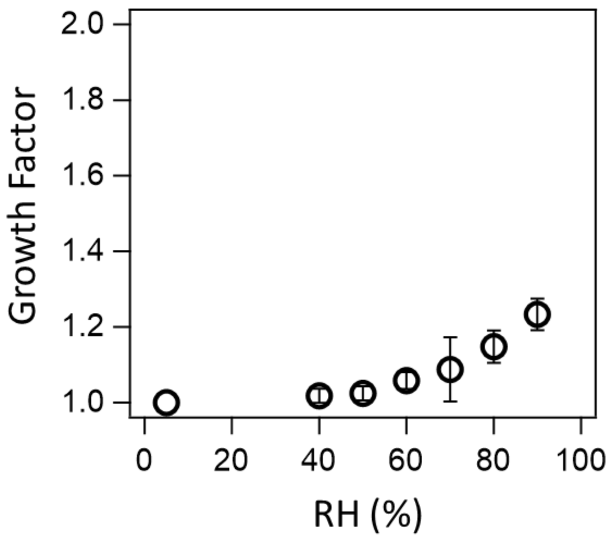

Fig. S23. The size growth factor of BBOA as a function of RH. The size factor denotes the size ratio of the wet particles at specific RHs to the dry particles, measured by the H-TDMA.

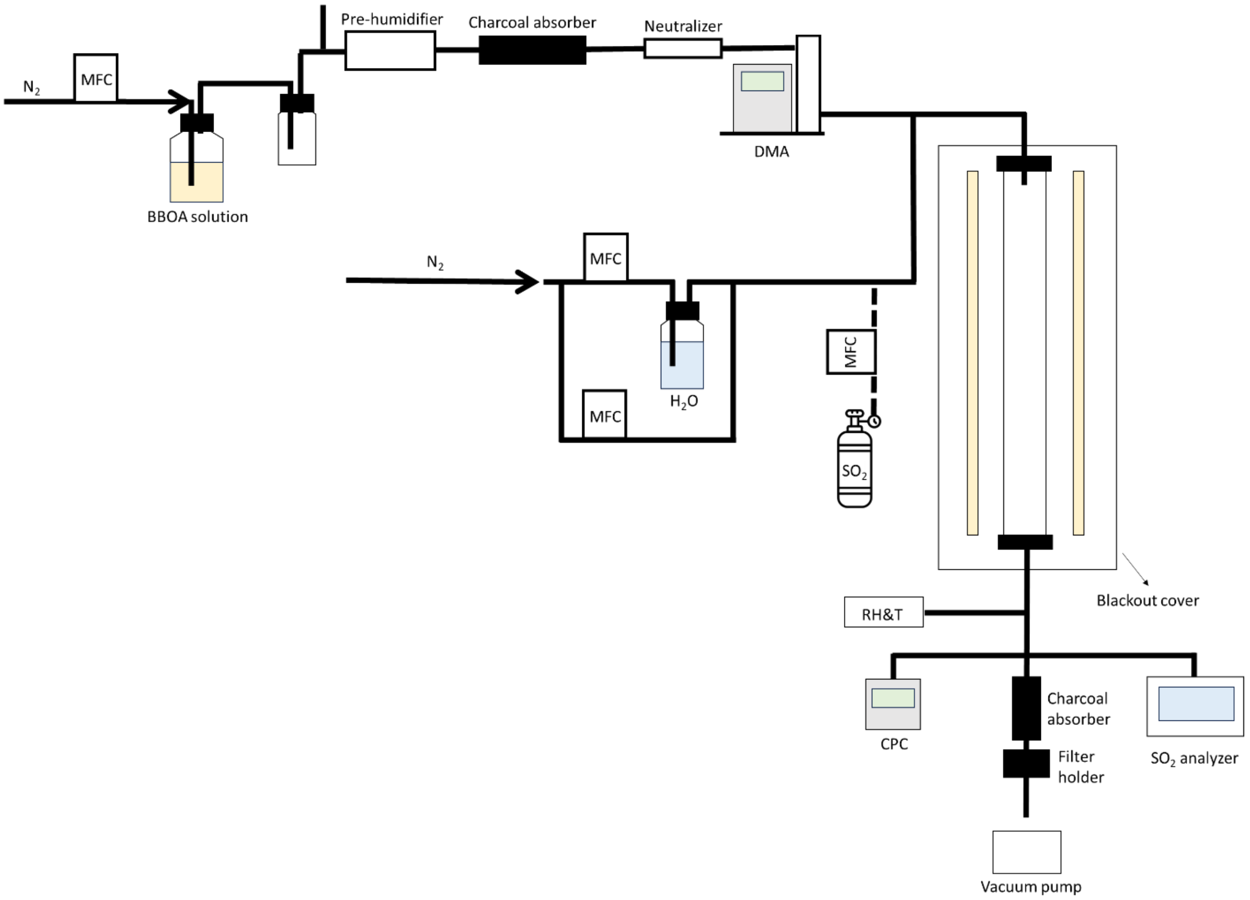

Fig. S24. The schematic of the aerosol flow tube. MFC denotes mass flow controller.

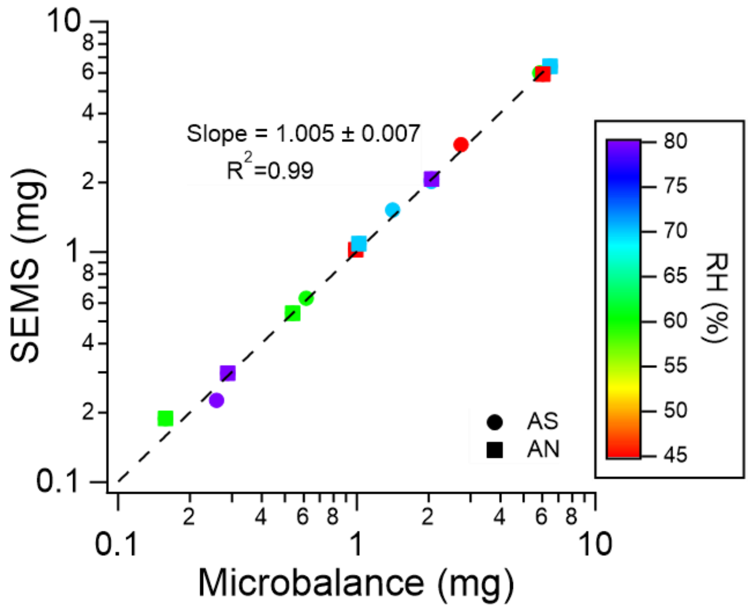

Fig. S25. Calibration of the particle mass loading measurements. The correlation between particle loading derived from Scanning Electrical Mobility Spectrometer (SEMS) measurements and that measured by the microbalance.

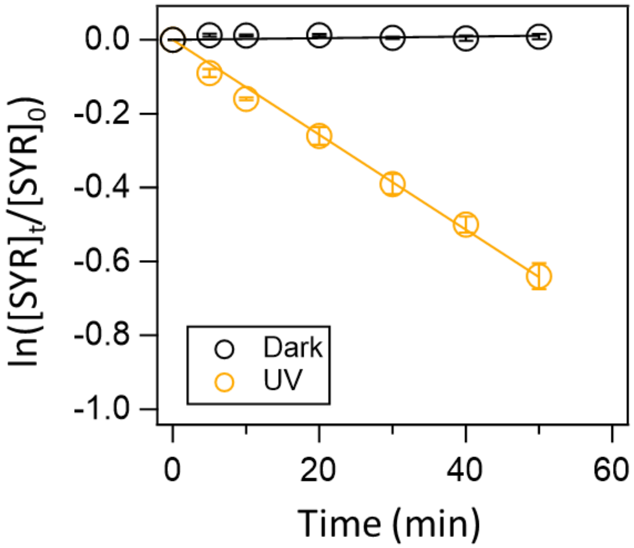

Fig. S26. The decay of syringol in BBOA solution. The logarithm of normalized syringol concentration as a function of time under dark and UV conditions.

Supplementary Tables

Table S1. The equilibrium constants for modeling the multiphase reactions.

Species	Equilibrium concentrations	Equilibrium constants	Reference
S(IV)	$[SO_2 \cdot H_2O] = H_{SO_2} \times P_{SO_2}$	$H_{SO_2} = 1.23 \times e^{(3145.3 \times (\frac{1}{T} - \frac{1}{298}))}$	(14)
	$[HSO_3^-] = K_{a1} \times \frac{[SO_2 \cdot H_2O]}{[H^+]}$	$K_{a1} = 1.3 \times 10^{-2} \times e^{(1960 \times (\frac{1}{T} - \frac{1}{298}))}$	
	$[SO_3^{2-}] = K_{a2} \times \frac{[HSO_3^-]}{[H^+]}$	$K_{a2} = 6.6 \times 10^{-8} \times e^{(1500 \times (\frac{1}{T} - \frac{1}{298}))}$	
H_2O_2	$[H_2O_2\ (aq)] = H_{H_2O_2} \times P_{H_2O_2}$	$H_{H_2O_2} = 1.3 \times 10^5 \times e^{(7297.1 \times (\frac{1}{T} - \frac{1}{298}))}$	

Table S2. The physicochemical parameters for different photosensitizers.

Category	Name	Structure	Formula	PDA (%)	Log K_{wa}[a]	Log K_{oa}[a]	Log K_{ow}[a]	Solubility ($M\ L^{-1}$)[a]	Volatility Regime[b]	E_T[c] ($kJ\ mol^{-1}$)	E^o (V.SHE)
Aromatic propenals	SinAld	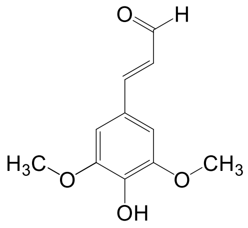	$C_{11}H_{12}O_4$	36.7	10.6	11.6	1.0	2.7×10^{-2}	IVOC	210	0.88
	ConAld	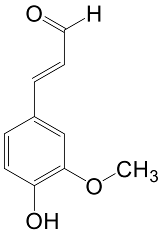	$C_{10}H_{10}O_3$	30.5	9.4	10.6	1.2	3.2×10^{-2}	IVOC	217	0.93

	Ferulic acid	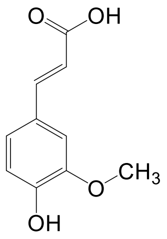	$C_{10}H_{10}O_4$	4.2	11.5	13.0	1.5	3.1×10^{-2}	IVOC	227	0.89
	Vanillylidene acetone	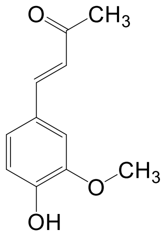	$C_{11}H_{12}O_3$	2.0	9.5	10.9	1.4	1.7×10^{-2}	IVOC	216	0.87
	4-methoxy-cinnamaldehyde	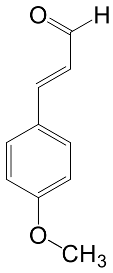	$C_{10}H_{10}O$	3.0	4.1	6.5	2.4	7.6×10^{-3}	IVOC	226	1.00
Aromatic Aldehyde	SyrAld	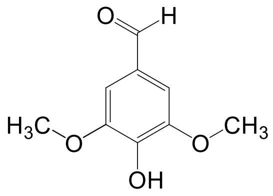	$C_9H_{10}O_4$	9.0	10.6	9.7	0.9	5.2×10^{-2}	IVOC	265	0.99

	DMB	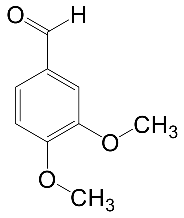	$C_9H_{10}O_3$	6.7	5.7	6.9	1.2	3.5×10^{-2}	IVOC	252	0.98
										173[d]	1.79[d]
	VL	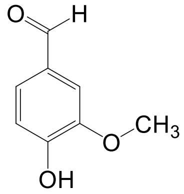	$C_8H_8O_3$	2.9	7.1	8.3	1.1	4.5×10^{-2}	IVOC	256	1.00
Coumarins	Nodakenetin	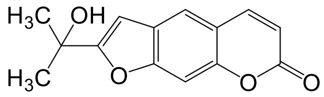	$C_{14}H_{14}O_4$	n.a.	9.2	11.0	1.8	8.5×10^{-3}	SVOC	254	1.05
Flavonoids	Apigenin	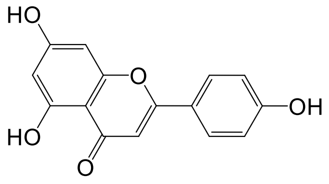	$C_{15}H_{10}O_5$	n.a.	14.7	17.7	3.0	6.8×10^{-4}	SVOC	261	1.27

PAHs	Pyrene	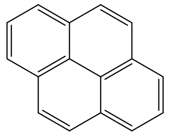	$C_{16}H_{10}$	n.a.	3.3	8.8	4.9	1.1×10^{-6}	IVOC	204	0.28
NACs	Methyl-hydroxy-dimethoxy-phenyl cyanate	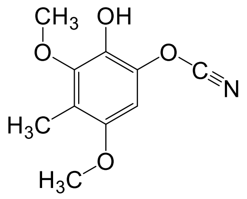	$C_{10}H_{11}O_4N$	n.a.	n.a.	n.a.	n.a.	n.a.	SVOC	n.a.	n.a.
	Methyl-hydroxy-timethoxy-phenyl cyanate	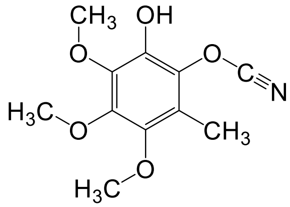	$C_{11}H_{13}O_5N$	n.a.	n.a.	n.a.	n.a.	n.a.	SVOC	n.a.	n.a.
Stilbenes	3,3-dimethoxy-4.4-dihydroxystilbene	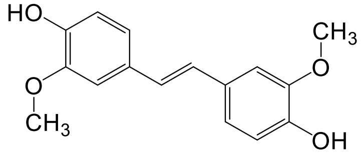	$C_{16}H_{16}O_4$	n.a.	n.a.	n.a.	n.a.	n.a.	SVOC	222	-0.02

[a]The data are sourced from Chem Spider.

[b]Estimated according to Li, Pöschl and Shiraiwa (34).

[c]Determined by $T_1 \rightarrow S_0$. The values are slightly different from those reported by Felber, Schaefer, He and Herrmann (35), which may be attributable to the different modeling settings.

[d]The protonated form. $pK_a(^3DMB^*)$ =3.3 (36).

Table S3. The parameters used for the estimation of the sulfate formation.

Location	Coordinates		BBOA	[$^3BBOA^*$]	[SO_2]	H_2O_2	Aerosol pH	Month
	Latitude	Longitude	(μg m^{-3})	(×10^{12} M^{-1})	(ppb)	(ppb)		
British Columbia, Canada	48 ~ 60	-139 ~ -114	10	1.25	0.11±0.12	0.33±0.08	0.73±0.40	2019 Jul
			40	1.05				
California, USA	32 ~ 42	-124 ~ -114	10	1.26	0.15±0.11	0.89±0.34	0.76±0.61	2019 Jul
			40	1.07				
Guizhou, China	24 ~ 29	103 ~ 109	10	1.29	1.00±0.63	0.98±0.19	2.35±0.12	2019 Jul
			40	1.10				
Amazonia, Brazil	-8 ~ -10	-70 ~ -63	10	1.16	0.08±0.05	1.28±0.16	1.37±0.19	2019 Jul
			40	0.94				
Leopoldville,	-5 ~ 14	12 ~ 31	10	1.26	0.27±0.89	1.22±0.40	1.53±0.34	2019 Jul

DR Congo			40	1.07				
Athens, Greece	23.5 ~ 24	37.5 ~ 38.5	10	1.30	1.25±0.70	1.04±0.02	-0.06±0.06	2019 Jul
			40	1.12				
Kalimantan, Indonesia	-4 ~ 7	108.5 ~ 119.5	10	1.24	0.19±0.10	1.14±0.37	1.23±0.53	2019 Jul
			40	1.04				
Siberia, Russia	52 ~ 56	113 ~ 118	10	1.13	0.24±0.15	0.47±0.06	1.66±0.35	2019 Jul
			40	0.90				
Darwin, Australia	-15 ~ -12	134 ~ 136	10	1.28	0.17±0.03	2.02±0.32	0.76±0.31	2019 Oct
			40	1.14				

Table S4. The sulfate formation rate under different conditions.

Particle type		Particle size (nm)	Light intensity (%)	$[SO_2]$ (ppb)	RH (%)	Measured $d[SO_4^{2-}]/dt$ ($M\ s^{-1}$)	Modeled $d[SO_4^{2-}]/dt$ ($M\ s^{-1}$)
BBOA	Particle	100	100	40	80	1.24×10^{-5}	4.06×10^{-9}
		100	50	40	80	7.44×10^{-6}	2.03×10^{-9}
		100	20	40	80	2.17×10^{-6}	8.12×10^{-10}
		200	100	40	80	6.22×10^{-6}	4.06×10^{-9}
		300	100	40	80	3.68×10^{-6}	4.06×10^{-9}
		100	100	70	80	2.33×10^{-5}	7.06×10^{-9}
		100	100	120	80	4.66×10^{-5}	1.21×10^{-8}
		100	100	40	70	1.26×10^{-5}	4.06×10^{-9}
		100	100	40	40	1.54×10^{-5}	4.06×10^{-9}

		100	0	40	80	3.48×10^{-7}	n.a.
		100	100	0	80	6.16×10^{-7}	n.a.
	Solution					4.40×10^{-6}	3.90×10^{-6}
DMB+buffer	Particle	100	100	40	88	3.36×10^{-5}	n.a.
	Solution					1.07×10^{-8}	n.a.
VL+buffer	Particle	100	100	40	88	3.26×10^{-6}	n.a.
	Solution					1.69×10^{-9}	n.a.
SyrAld+buffer	Particle	100	100	40	88	3.10×10^{-6}	n.a.
	Solution					9.81×10^{-10}	n.a.
ConAld+buffer	Particle	100	100	40	88	5.49×10^{-7}	n.a.
	Solution					4.78×10^{-10}	n.a.
SinAld+buffer	Particle	100	100	40	88	7.78×10^{-7}	n.a.

	Solution					6.35×10^{-10}	n.a.
2-IC+buffer	Particle	100	100	40	88	1.35×10^{-5}	n.a.
	Solution					6.35×10^{-9}	n.a.

Table S5. The kinetic parameters for estimating the $^3BBOA^*$ concentration.

Parameter	Method	pH	Value
F_T (M C s^{-1})	Chemical probing	1	1.0×10^{-4} [OM]
		4	1.2×10^{-4} [OM]
k_{OM}[b] (M^{-1} C s^{-1})	Chemical probing	1	2.9×10^{8}
		4	2.4×10^{8}
$k_{S(IV)}$ (M^{-1} s^{-1})	TA[a]	1	6.0×10^{8}
		4	5.1×10^{8}
		7	2.9×10^{8}
k_{O_2} (M^{-1} s^{-1})	TA[a]	1	1.3×10^{9}
		4	1×10^{9}

[a]It should be noted that TA measured the total quenching rate constants including reactive and non-reactive.

Reference

1. Z. Liang, Y. Chu, M. Gen, C. K. Chan, Single-particle Raman spectroscopy for studying physical and chemical processes of atmospheric particles. *Atmospheric Chemistry and Physics* **22**, 3017-3044 (2022).
2. M. Li, Y. Kan, H. Su, U. Pöschl, S. H. Parekh, M. Bonn, Y. Cheng, Spatial homogeneity of pH in aerosol microdroplets. *Chem* **9**, 1036-1046 (2023).
3. M. Li, H. Su, G. Zheng, U. Kuhn, N. Kim, G. Li, N. Ma, U. Pöschl, Y. Cheng, Aerosol pH and ion activities of HSO_4^- and SO_4^{2-} in supersaturated single droplets. *Environmental Science & Technology* **56**, 12863-12872 (2022).
4. H. J. Lin, P. Herman, J. R. Lakowicz, Fluorescence lifetime‐resolved pH imaging of living cells. *Cytometry part A: the journal of the international society for analytical cytology* **52**, 77-89 (2003).
5. R. L. Craig, P. K. Peterson, L. Nandy, Z. Lei, M. A. Hossain, S. Camarena, R. A. Dodson, R. D. Cook, C. S. Dutcher, A. P. Ault, Direct Determination of Aerosol pH: Size-Resolved Measurements of Submicrometer and Supermicrometer Aqueous Particles. *Analytical Chemistry* **90**, 11232-11239 (2018).
6. G. Li, H. Su, N. Ma, G. Zheng, U. Kuhn, M. Li, T. Klimach, U. Pöschl, Y. Cheng, Multifactor colorimetric analysis on pH-indicator papers: an optimized approach for direct determination of ambient aerosol pH. *Atmospheric Measurement Techniques Discussions* **2019**, 1-19 (2019).
7. L. Ma, R. Worland, W. Jiang, C. Niedek, C. Guzman, K. J. Bein, Q. Zhang, C. Anastasio, Predicting photooxidant concentrations in aerosol liquid water based on laboratory extracts of ambient particles. *Atmospheric Chemistry and Physics* **23**, 8805-8821 (2023).
8. L. Ashton, G. V. Buxton, C. R. Stuart, Temperature dependence of the rate of reaction of OH with some aromatic compounds in aqueous solution. Evidence for the formation of a π-complex intermediate? *Journal of the Chemical Society, Faraday Transactions* **91**, 1631-1633 (1995).
9. T. Lu, F. Chen, Multiwfn: A multifunctional wavefunction analyzer. *Journal of computational chemistry* **33**, 580-592 (2012).
10. F. Santoro, D. Jacquemin, Going beyond the vertical approximation with time-dependent density functional theory. *WIREs Computational Molecular Science* **6**, 460-486 (2016).
11. A. V. Marenich, C. J. Cramer, D. G. Truhlar, Universal Solvation Model Based on Solute Electron Density and on a Continuum Model of the Solvent Defined by the Bulk Dielectric Constant and Atomic Surface Tensions. *The Journal of Physical Chemistry B* **113**, 6378-6396 (2009).

12. J. Gao, H. Wang, W. Liu, H. Xu, Y. Wei, X. Tian, Y. Feng, S. Song, G. Shi, Hydrogen peroxide serves as pivotal fountainhead for aerosol aqueous sulfate formation from a global perspective. *Nature Communications* **15**, 4625 (2024).

13. A. K. Lee, M. D. Willis, R. M. Healy, J. M. Wang, C.-H. Jeong, J. C. Wenger, G. J. Evans, J. P. Abbatt, Single-particle characterization of biomass burning organic aerosol (BBOA): evidence for non-uniform mixing of high molecular weight organics and potassium. *Atmospheric Chemistry and Physics* **16**, 5561-5572 (2016).

14. J. H. Seinfeld, S. N. Pandis, *Atmospheric chemistry and physics: from air pollution to climate change* (John Wiley & Sons, 2016).

15. L. I. Kleinman, A. J. Sedlacek Iii, K. Adachi, P. R. Buseck, S. Collier, M. K. Dubey, A. L. Hodshire, E. Lewis, T. B. Onasch, J. R. Pierce, J. Shilling, S. R. Springston, J. Wang, Q. Zhang, S. Zhou, R. J. Yokelson, Rapid evolution of aerosol particles and their optical properties downwind of wildfires in the western US. *Atmos. Chem. Phys.* **20**, 13319-13341 (2020).

16. D. Richardson, A. S. Black, D. Irving, R. J. Matear, D. P. Monselesan, J. S. Risbey, D. T. Squire, C. R. Tozer, Global increase in wildfire potential from compound fire weather and drought. *NPJ climate and atmospheric science* **5**, 23 (2022).

17. A. Ansmann, K. Ohneiser, A. Chudnovsky, D. A. Knopf, E. W. Eloranta, D. Villanueva, P. Seifert, M. Radenz, B. Barja, F. Zamorano, C. Jimenez, R. Engelmann, H. Baars, H. Griesche, J. Hofer, D. Althausen, U. Wandinger, Ozone depletion in the Arctic and Antarctic stratosphere induced by wildfire smoke. *Atmos. Chem. Phys.* **22**, 11701-11726 (2022).

18. S. Solomon, K. Dube, K. Stone, P. Yu, D. Kinnison, O. B. Toon, S. E. Strahan, K. H. Rosenlof, R. Portmann, S. Davis, On the stratospheric chemistry of midlatitude wildfire smoke. *Proceedings of the National Academy of Sciences* **119**, e2117325119 (2022).

19. A. Hodzic, S. Madronich, B. Bohn, S. Massie, L. Menut, C. Wiedinmyer, Wildfire particulate matter in Europe during summer 2003: meso-scale modeling of smoke emissions, transport and radiative effects. *Atmospheric Chemistry and Physics* **7**, 4043-4064 (2007).

20. X. Ye, M. Deshler, A. Lyapustin, Y. Wang, S. Kondragunta, P. Saide, Assessment of Satellite AOD during the 2020 Wildfire Season in the Western U.S. *Remote Sensing* **14**, 6113 (2022).

21. A. P. Williams, J. T. Abatzoglou, A. Gershunov, J. Guzman‐Morales, D. A. Bishop, J. K. Balch, D. P. Lettenmaier, Observed impacts of anthropogenic climate change on wildfire in California. *Earth's Future* **7**, 892-910 (2019).

22. Z. Xue, P. Gupta, S. Christopher, Satellite-based estimation of the impacts of summertime wildfires on $PM_{2.5}$ concentration in the United States. *Atmos. Chem. Phys.* **21**, 11243-11256 (2021).

23. X. Zhang, K. Gui, T. Liao, Y. Li, X. Wang, X. Zhang, H. Ning, W. Liu, J. Xu, Three-dimensional spatiotemporal evolution of wildfire-induced smoke aerosols: A case study from Liangshan, Southwest China. *Science of The Total Environment* **762**, 144586 (2021).

24. V. Selimovic, *Air Quality and Climate Impacts of Western US Wildfires* (University of Montana, 2020).

25. S. Bajocco, G. B. Pezzatti, S. Mazzoleni, C. Ricotta, Wildfire seasonality and land use: when do wildfires prefer to burn? *Environmental monitoring and assessment* **164**, 445-452 (2010).

26. E. S. Galbavy, K. Ram, C. Anastasio, 2-Nitrobenzaldehyde as a chemical actinometer for solution and ice photochemistry. *Journal of Photochemistry and Photobiology A: Chemistry* **209**, 186-192 (2010).

27. Y. Wang, T. Qiu, C. Zhang, T. Hao, B. R. G. Mabato, R. Zhang, M. Gen, M. N. Chan, D. D. Huang, X. Ge, Co-photolysis of mixed chromophores affects atmospheric lifetimes of brown carbon. *Environmental Science: Atmospheres* **3**, 1145-1158 (2023).

28. J. D. Smith, H. Kinney, C. Anastasio, Phenolic carbonyls undergo rapid aqueous photodegradation to form low-volatility, light-absorbing products. *Atmospheric Environment* **126**, 36-44 (2016).

29. X. Wang, R. Gemayel, N. Hayeck, S. Perrier, N. Charbonnel, C. Xu, H. Chen, C. Zhu, L. Zhang, L. Wang, S. A. Nizkorodov, X. Wang, Z. Wang, T. Wang, A. Mellouki, M. Riva, J. Chen, C. George, Atmospheric Photosensitization: A New Pathway for Sulfate Formation. *Environmental Science & Technology* **54**, 3114-3120 (2020).

30. Y. Lyu, Y. H. Lam, Y. Li, N. Borduas-Dedekind, T. Nah, Seasonal variations in the production of singlet oxygen and organic triplet excited states in aqueous $PM_{2.5}$ in Hong Kong SAR, South China. *Atmos. Chem. Phys.* **23**, 9245-9263 (2023).

31. R. Kaur, J. R. Labins, S. S. Helbock, W. Jiang, K. J. Bein, Q. Zhang, C. Anastasio, Photooxidants from brown carbon and other chromophores in illuminated particle extracts. *Atmospheric Chemistry and Physics* **19**, 6579-6594 (2019).

32. M. He, J. C. Ditto, L. Gardner, J. Machesky, T. N. Hass-Mitchell, C. Chen, P. Khare, B. Sahin, J. D. Fortner, D. L. Plata, Total organic carbon measurements reveal major gaps in petrochemical emissions reporting. *Science* **383**, 426-432 (2024).

33. Q. Bian, S. H. Jathar, J. K. Kodros, K. C. Barsanti, L. E. Hatch, A. A. May, S. M. Kreidenweis, J. R. Pierce, Secondary organic aerosol formation in biomass-burning plumes: theoretical analysis of lab studies and ambient plumes. *Atmospheric Chemistry and Physics* **17**, 5459-5475 (2017).

34. Y. Li, U. Pöschl, M. Shiraiwa, Molecular corridors and parameterizations of volatility in the chemical evolution of organic aerosols. *Atmospheric Chemistry and Physics* **16**, 3327-3344 (2016).

35. T. Felber, T. Schaefer, L. He, H. Herrmann, Aromatic Carbonyl and Nitro Compounds as Photosensitizers and Their Photophysical Properties in the Tropospheric Aqueous Phase. *The Journal of Physical Chemistry A* **125**, 5078-5095 (2021).

36. J. D. Smith, V. Sio, L. Yu, Q. Zhang, C. Anastasio, Secondary Organic Aerosol Production from Aqueous Reactions of Atmospheric Phenols with an Organic Triplet Excited State. *Environmental Science & Technology* **48**, 1049-1057 (2014).
